# Factorization by quantum annealing using superconducting flux qubits implementing a multiplier Hamiltonian

**DOI:** 10.1038/s41598-022-17867-9

**Published:** 2022-08-11

**Authors:** Daisuke Saida, Mutsuo Hidaka, Kentaro Imafuku, Yuki Yamanashi

**Affiliations:** 1 Device Research Institute, National Institute of Advanced Industrial Science and Technology, Central2, 1-1-1 Umezono, Tsukuba, Ibaraki 305-8568, Japan; 2 Quantum laboratory, Fujitsu Limited, 1-1, Kamikodanaka 4-chome, Nakahara-ku, Kawasaki, Kanagawa 211-8588, Japan; 3 School of Engineering Science, Yokohama National University, 79-5 Tokiwadai, Hodogaya, Yokohama, Kanagawa 240-8501, Japan

**Keywords:** Electrical and electronic engineering, Electronics, photonics and device physics, Quantum physics

## Abstract

Prime factorization (*P* = *M* × *N*) is a promising application for quantum computing. Shor’s algorithm is a key concept for breaking the limit for analyzing *P*, which cannot be effectively solved by classical computation; however, the algorithm requires error-correctable logical qubits. Here, we describe a quantum annealing method for solving prime factorization. A superconducting quantum circuit with native implementation of the multiplier Hamiltonian provides combinations of *M* and *N* as a solution for number *P* after annealing. This circuit is robust and can be expanded easily to scale up the analysis. We present an experimental and theoretical exploration of the multiplier unit. We demonstrate the 2-bit factorization in a circuit simulation and experimentally at 10 mK. We also explain how the current conditions can be used to obtain high success probability and all candidate factorized elements.

## Introduction

Quantum computing is a computational method that provides an alternative route to solving problems that classical computing cannot solve effectively^1^. Experimental demonstrations of quantum computing, using nuclear magnetic resonance^2^, trapped ions^3,4^, photons^5^, and superconducting qubits^6–8^, have been reported. A simpler architecture for quantum computation is quantum annealing (QA), which provides a more practical approach in the near-term^9–18^. QA uses a Hamiltonian that expresses the problems to be solved with a time-dependent term for initializing the ground state. At the end of the evolution, the ground state represents the lowest energy configuration for the problem Hamiltonian, and thus a solution to the optimization problem^14,19–26^. We have proposed QA with native implementation of the Hamiltonian in a superconducting quantum circuit^24^. The problem Hamiltonian, which has a set of ground states consistent with a given truth table, is implemented for the circuit with no redundant qubits. This direct implementation of the original Hamiltonian is vital for obtaining solutions with high success probability because the original energy relationship in the Hamiltonian is preserved^24,25^.

**Figure 1:**
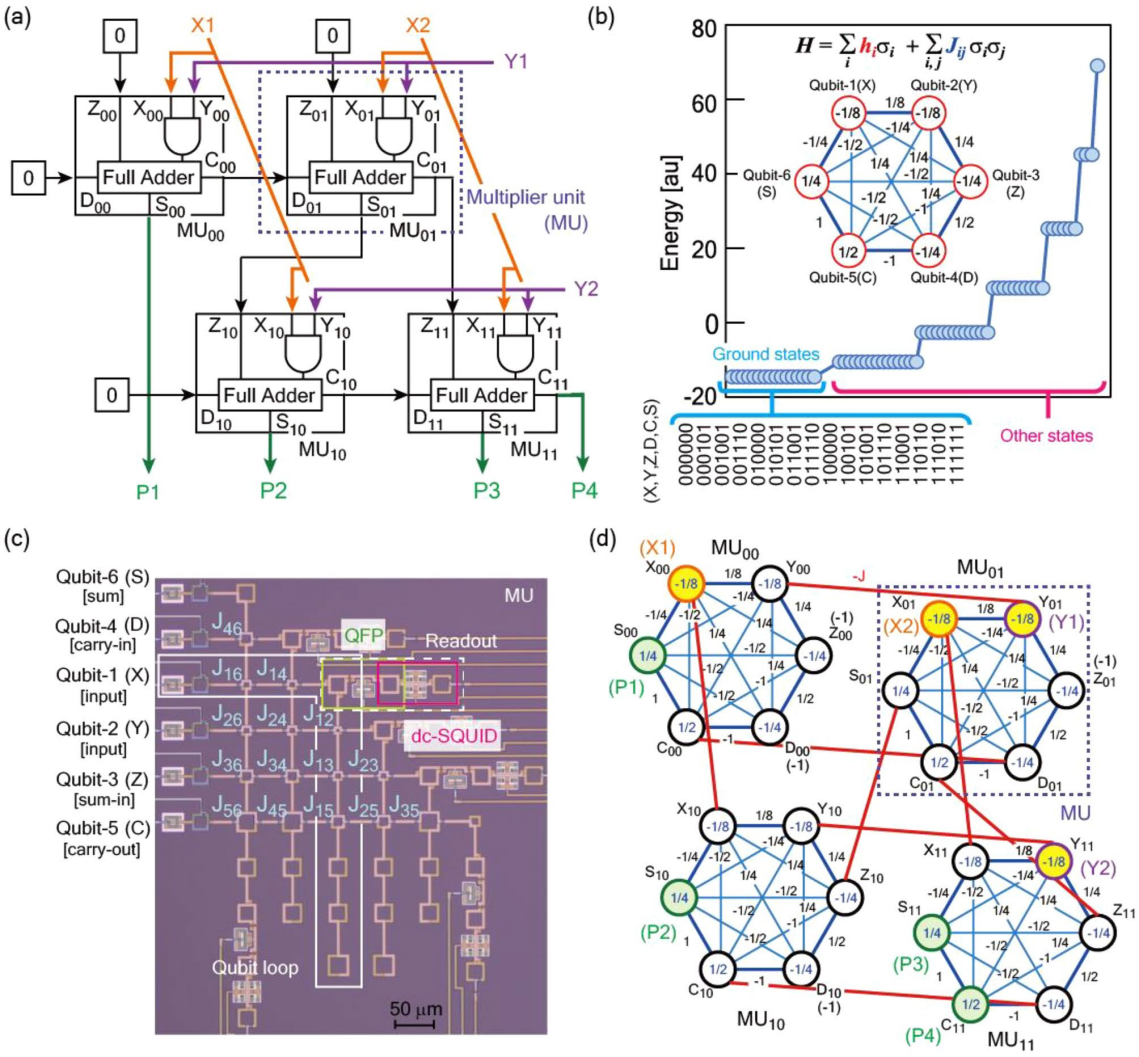
Scalable factorization circuit and its key component, the multiplier unit. (**a**) Schematic of classical 4-bit multiplier. This consists of four multiplier unit (MU) indicated by dotted rectangle. (**b**) Energy of each state in the MU. *J*_*ij*_ terms of the Hamiltonian are implemented by tuning the overlapping area between qubits *i* and *j*. After quantum annealing, each qubit state takes combinations to reach the Hamiltonian minimum energy. (**c**) A superconducting quantum circuit for the MU consisting of six superconducting flux qubits with all-to-all connectivity. The qubit state is detected by a readout circuit composed of a quantum flux parametron (QFP) and a dc superconducting quantum interference device (SQUID). (**d**) Description of Hamiltonian in the 4-bit multiplier. Production can be described as *P* = (*P*_4_ *P*_3_ *P*_2_ *P*_1_)_(2)_ = (C_11_ S_11_ S_10_ S_00_)_(2)_. Inputs are represented as *M* = (*X*_2_ *X*_1_)_(2)_ = (X_01_ X_00_)_(2)_ and *N* = (*Y*_2_ *Y*_1_)_(2)_ = (Y_11_ Y_01_)_(2)_, respectively. Superconducting quantum circuit embedding this Hamiltonian can provide function of 4-bit prime factorization in QA.

Prime factorization (*P* = *M* × *N*) is a promising application for quantum computing^20,27,28^. The development of Shor’s algorithm stimulated intense interest in quantum computing^29^. However, error-correctable qubits are required to implement the algorithm^6–8,30^. For accurate error corrections, the fabrication of millions to billions of qubits is challenging. Another candidate method uses QA, where the prime factorization is treated as an optimization problem with solutions as the global minimum of the Hamiltonian^20^. However, in this method, classical computation is required to calculate the Gröbner basis, which helps to reduce the cost function. We have proposed an alternative method that can solve the prime factorization by QA directly. The method uses a superconducting quantum circuit of a multiplier, which provides combinations of *M* and *N* as a solution after QA when number *P* is set initially^24–26^. Basic idea is based on use of a classical *n*-bit multiplier^31,32^. We define “*n*-bit” corresponds to the length of bit string *P*. Figure 1a shows the classical 4-bit multiplier. The element of the multiplier; named as a multiplier unit (MU), is built from the function of AND processing and the full adder gate (surrounded by dotted rectangle). The 4-bit multiplier is composed of four MU_*ij*_ (*i* = 0–1, *j* = 0–1). The MU_*ij*_ consists of *X*_*ij*_ and *Y*_*ij*_ for inputs, *Z*_*ij*_ for sum-in, *D*_*ij*_ for carry-in, *C*_*ij*_ for carry-out, and *S*_*ij*_ for summation. Production of this multiplier can be described as *P* = (*P*_4_ *P*_3_ *P*_2_ *P*_1_)_(2)_ = (C_11_ S_11_ S_10_ S_00_)_(2)_. Inputs are represented as *M* = (*X*_2_ *X*_1_)_(2)_ = (X_01_ X_00_)_(2)_ and *N* = (*Y*_2_ *Y*_1_)_(2)_ = (Y_11_ Y_01_)_(2)_, respectively. Here, a binary number representation is utilized. The input of *D*_*01*_ can be fixed to 0, since *D*_*01*_ is 0 regardless of *X*_*1*_ and *Y*_*1*_. The MU shown in Fig. 1a is flexible enough to adapt both inputs of fixed 0 and digit up from the previous step. Using the ground state spin logic^33^, the classical MU can be expressed as a Hamiltonian shown in Fig. 1b ^24,25^. The Hamiltonian of the MU consists of six superconducting flux qubits. Qubit-1 and -2 corresponds to the inputs. Qubit-3 and -4 are carry-in. Qubit-5 and -6 behaves carry-out and summation, respectively. Our unique method is direct implementation of the Hamiltonian to the superconducting quantum circuit using static magnetic coupling shown in Fig. 1c. Figure 1d shows description of the Hamiltonian of the 4-bit multiplier. Transportations of carries in the classical multiplier corresponds to interaction between qubits in the superconducting quantum circuit. Four qubits of C_11_, S_11_, S_10_, S_00_ are components of the product *P* in the multiplications (green circles shown in Fig. 1d). Pairs of two qubits (X_01_ & X_00_, Y_11_ & Y_01_) corresponds to the inputs (represented as orange circles for *M* and purple circles for *N* in Fig. 1d). Note that this circuit does not calculate each MU step by step with taking account of carry flow like classical multiplier. Combinations of qubit sates that has minimum energy, corresponding to the ground states of the Hamiltonian, occur after the QA. To take advantage of this property, we design the superconducting circuit where the ground states are aligned with the desired truth table. This circuit has function of an invertible logic^34^ with the multiplication and the factorization. The superconducting quantum circuit for the prime factorization could be extended easily by increasing the number of MUs. In this letter and a related study, we examine the MU composed of six superconducting flux qubits. We demonstrate an MU prototype in Ref.^24^; however, this letter focuses on the theoretical and experimental behavior of the MU by considering the qubit configurations. We investigate a method that can improve the accuracy of solutions and obtain all candidate factorized elements. We also demonstrate the robustness of the operating bias conditions in the MU and the promise of scaling for prime factorization.

## Results

### Ideal behavior in the MU

**Figure 2:**
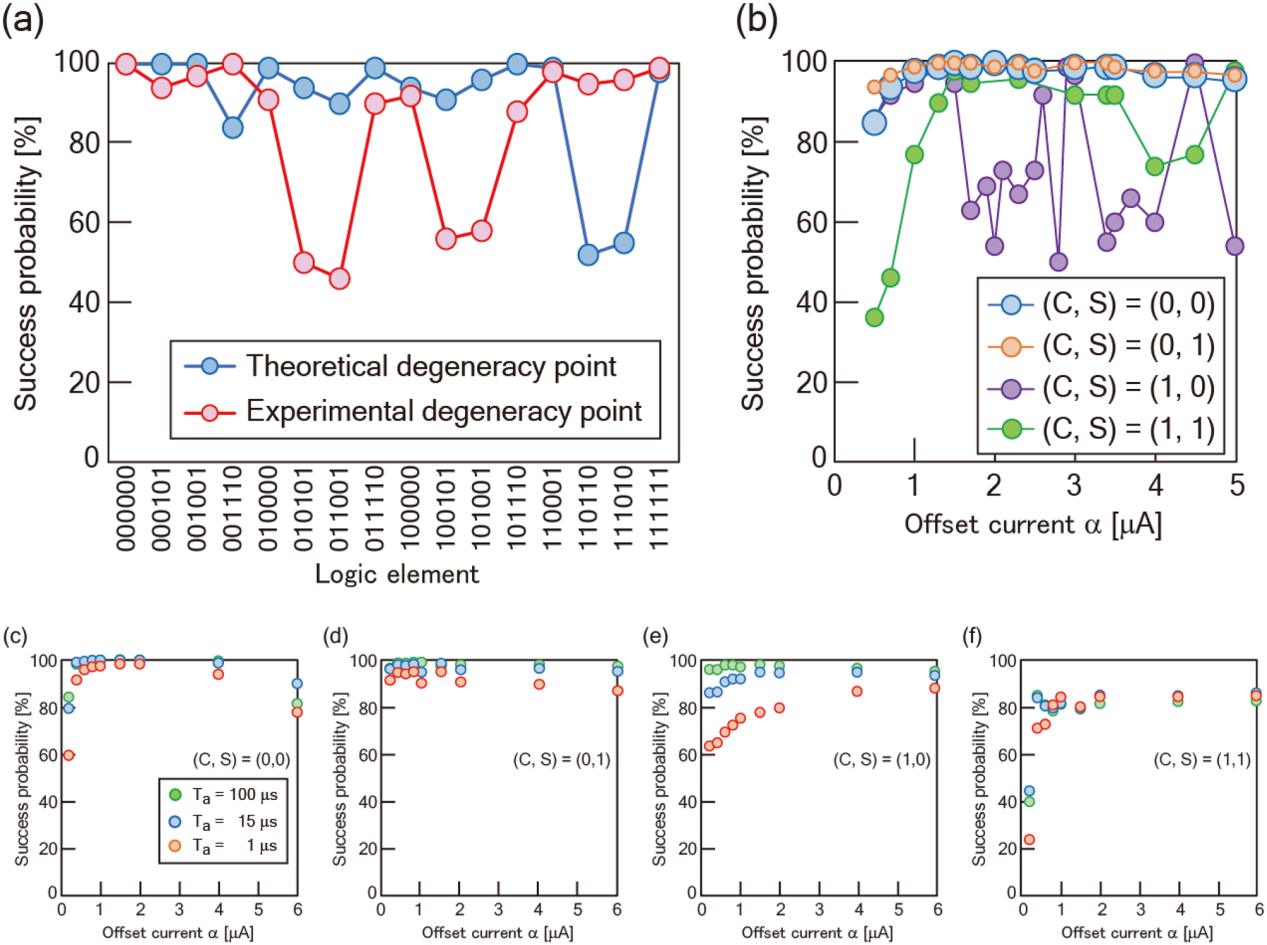
Demonstration of the multiplication and the factorization. (**a**) Success probabilities of the multiplication calculated by JSIM analysis based on two kinds of degeneracy points in MU2. The theoretical degeneracy point is the current conditions numerically estimated using Eq. (1). The experimental degeneracy point is the current conditions where all 16 logic components are observed. An offset current α of 1.3 μA is used. All candidate logic components are reproduced in both analyses; however, the probability is affected by the applied degeneracy point. (**b**) α dependence of success probabilities of the factorization calculated by JSIM analysis in MU2. Factorizations of (1,0)_(2)_ and (1,1)_(2)_ are not stable with respect to the increase of α. α dependence of the experimental success probabilities of the factorization in (**c**) (0,0)_(2)_, (**d**) (0,1)_(2)_, (**e**) (1,0)_(2)_, and (**f**) (1,1)_(2)_. Longer annealing time (*T*_a_) increases the success probability.

The Hamiltonian in Fig. 1b is directly implemented in the MU (Fig. 1c). Our designed instances are MUs composed of the six qubits with all-to-all connectivity at critical currents (*I*_c_) of 6.25 μA (MU1) and 3.75 μA (MU2). The sample configuration is described in the “Methods” section. Sixteen combinations of (*X*, *Y*, *Z*, *D*, *C*, *S*), which correspond to logic components in the MU, take the minimum energy. These combinations appear at a degeneracy point after QA. Theoretically, considering the MU Hamiltonian, the degeneracy point is expressed as

1 $$\begin{aligned} & I_{{h2}} = I_{{h1}} , \\ & I_{{h4}} = I_{{h3}} = \frac{{M_{1} }}{{M_{3} }} \cdot \frac{{M_{{13}} }}{{M_{{12}} }} \cdot I_{{h1}} , \\ & I_{{h5}} = \frac{{M_{1} }}{{M_{5} }} \cdot \frac{{M_{{15}} }}{{M_{{12}} }} \cdot I_{{h1}} , \\ & I_{{h6}} = \frac{{M_{1} }}{{M_{6} }} \cdot \frac{{M_{{16}} }}{{M_{{12}} }} \cdot I_{{h1}} , \\ \end{aligned}$$

where *I*_h*i*_ (*i* = 1–6) is the external bias of qubit *i*, *M*_*i*_ (*i* = 1–6) is the mutual inductance between qubit *i* and the external bias line, and *M*_*ij*_ (*i* = 1–6, *j* = 1–6) is the mutual inductance between qubits *i* and *j*. The elicitation process of Eq. (1) is described in the “Supplementary Methods”. The inductances of qubits and mutual inductances are extracted from the MU layout (see “Methods”). The degeneracy points of MU1 and MU2 are estimated as (*I*_h1_, *I*_h2_, *I*_h3_, *I*_h4_, *I*_h5_, *I*_h6_) = (− 0.4, − 0.4, − 0.8, − 0.8, 1.6, 0.8) and (− 0.3, − 0.3, − 0.6, − 0.6, 1.2, 0.6) [μA], respectively. We can produce a desirable logic component by applying an appropriate offset current, α, against the degeneracy point. For example, multiplication of (*X*, *Y*, *Z*, *D*) = (1, 1, 0, 0), where carry-ins are fixed as 0, can be considered by applying the external flux bias of (*I*_h1_′, *I*_h2_′, *I*_h3_′, *I*_h4_′, *I*_h5_′, *I*_h6_′) = (*I*_h1_ + α, *I*_h2_ + α, *I*_h3_ − α, *I*_h4_ − α, *I*_h5_, *I*_h6_). After QA, each qubit state takes combinations to reach the minimum energy in the Hamiltonian. Biasing one of the qubits by adopting α in the initial condition restricts the state of the other qubit because the qubits interact with each other to minimize the energy after QA. In the multiplication of (*X*, *Y*, *Z*, *D*) = (1, 1, 0, 0), qubit-5 (C) and qubit-6 (S) probably adopt the 0 and 1 states, respectively, because the combinations of qubits (*X*, *Y*, *Z*, *D, C, S*) = (1, 1, 0, 0, 0, 1) produces the minimum energy. Based on the theoretically evaluated degeneracy point, we examine the multiplication with a Josephson integrated circuit simulation (JSIM)^35^ (see “Methods” section). Figure 2a shows all candidate multiplications of each logic component in the simulation. The 16 combinations of qubits (*X*, *Y*, *Z*, *D, C, S*), corresponding to the logic components in the MU, are all reproduced. Except from (*X*, *Y*, *Z*, *D, C, S*) = (1, 1, 0, 1, 1, 0) and (1, 1, 1, 0, 1, 0), the success probabilities are above 80%. Multiplications are also simulated using the degeneracy points, which are obtained experimentally. Although the elements with low success probabilities varies, all components of the multiplication are reproduced. In the factorization process, α is assigned to qubits (*C, S*). The MU covers the numbers 3_(10)_ = (1,1)_(2)_, 2_(10)_ = (1,0)_(2)_, 1_(10)_ = (0,1)_(2)_, 0_(10)_ = (0,0)_(2)_. For example, factorization of 2_(10)_ is performed by applying the external flux bias of (*I*_h1_′, *I*_h2_′, *I*_h3_′, *I*_h4_′, *I*_h5_′, *I*_h6_′) = (*I*_h1_, *I*_h2_, *I*_h3_, *I*_h4_, *I*_h5_ + α, *I*_h6_ − α). Due to the global minima restriction in the Hamiltonian, combinations of qubit states probably produce five candidates, which are (*X*, *Y*, *Z*, *D, C, S*) = (0, 0, 1, 1, 1, 0), (0, 1, 1, 1, 1, 0), (1, 0, 1, 1, 1, 0), (1, 1, 0, 1, 1, 0), and (1, 1, 1, 0, 1, 0). Figure 2b represents the JSIM analysis of the factorization using the theoretical degeneracy point. The success probabilities in the factorization of number 1_(10)_ and 0_(10)_ increase as α increases, later slightly decreases. In contrast, the factorization of numbers 2_(10)_ and 3_(10)_ show different behavior, where the success probability obviously decreases above α of 1.3 and 2.5 μA, respectively. This indicates that there is an optimum value of α, and the highest success probability is obtained with α of 1.3 μA.

### Hamiltonian implementation

Direct implementation of the Hamiltonian produces a ground state in the superconducting quantum circuit at the minimum energy. We should satisfy two requirements for direct implementation, based on the relationships of the qubit-to-qubit interactions (corresponding to *M*_*ij*_) and qubit-to-local current path interactions (corresponding to mutual inductances *M*_*i*_). The elicitation process is described in the “Supplementary Methods”. The designs of MU1 and MU2 generally satisfy the two requirements, as shown in Supplementary Tables S1 and S2.

### Degeneracy point

The superconducting quantum circuits are cooled to 10 mK in a dilution refrigerator. Under these conditions, the energy in the potential of an rf superconducting quantum interference device (SQUID) at the qubit is four orders of magnitude higher than that of the thermal energy. Disturbance due to the thermal energy is neglectable in our experiment. The electric noise is sufficiently suppressed so that we can evaluate a switching current of 0.28 μA in a single Josephson junction, which is much less than *I*_c_ of the qubit in this work (see Supplementary Fig. S1c). Based on the experiment described in “Experimental configurations” of the “Supplementary Methods”, the experimental degeneracy points of MU1 and MU2 are found at (*I*_h1_, *I*_h2_, *I*_h3_, *I*_h4_, *I*_h5_, *I*_h6_) of (− 0.28, − 0.23, − 0.45, − 0.6, 0.37, 0.42) and (− 0.3, − 0.3, − 0.4, − 0.4, 0.5, 0.4) [μA], respectively (see Supplementary Fig. S2). For convenience, we define these current conditions as OP1 and OP2, respectively. OP1 and OP2 are different from the theoretically estimated current conditions. Annealing time (*T*_a_) dependence appears, especially in frequently generated elements and minor elements. The frequency of occurrence of each element in MU2 is more uniform than that in MU1. The similarity in the trends in the frequency of other elements (error response) and in frequently generated elements are because configuration of the superconducting quantum circuits are the same in MU1 and MU2. The frequency of the minor elements in MU2 is improved compared with that in MU1. Although the theoretical and experimental degeneracy points are different, we can use these conditions to generate the logic components in the multiplication and factorization with α. A typical response of the multiplication in MU1 based on OP1 is reported in Ref.^24^. Supplementary Fig. S3 shows an example of the multiplication using OP2. All 16 elements in Fig. 1b are reproduced, and the multiplication of (*X*, *Y*, *Z*, *D*) = (1, 1, 1, 1) (Supplementary Fig. S3a) shows that there is an appropriate α value for generating states with high accuracy.

### Factorization

**Figure 3:**
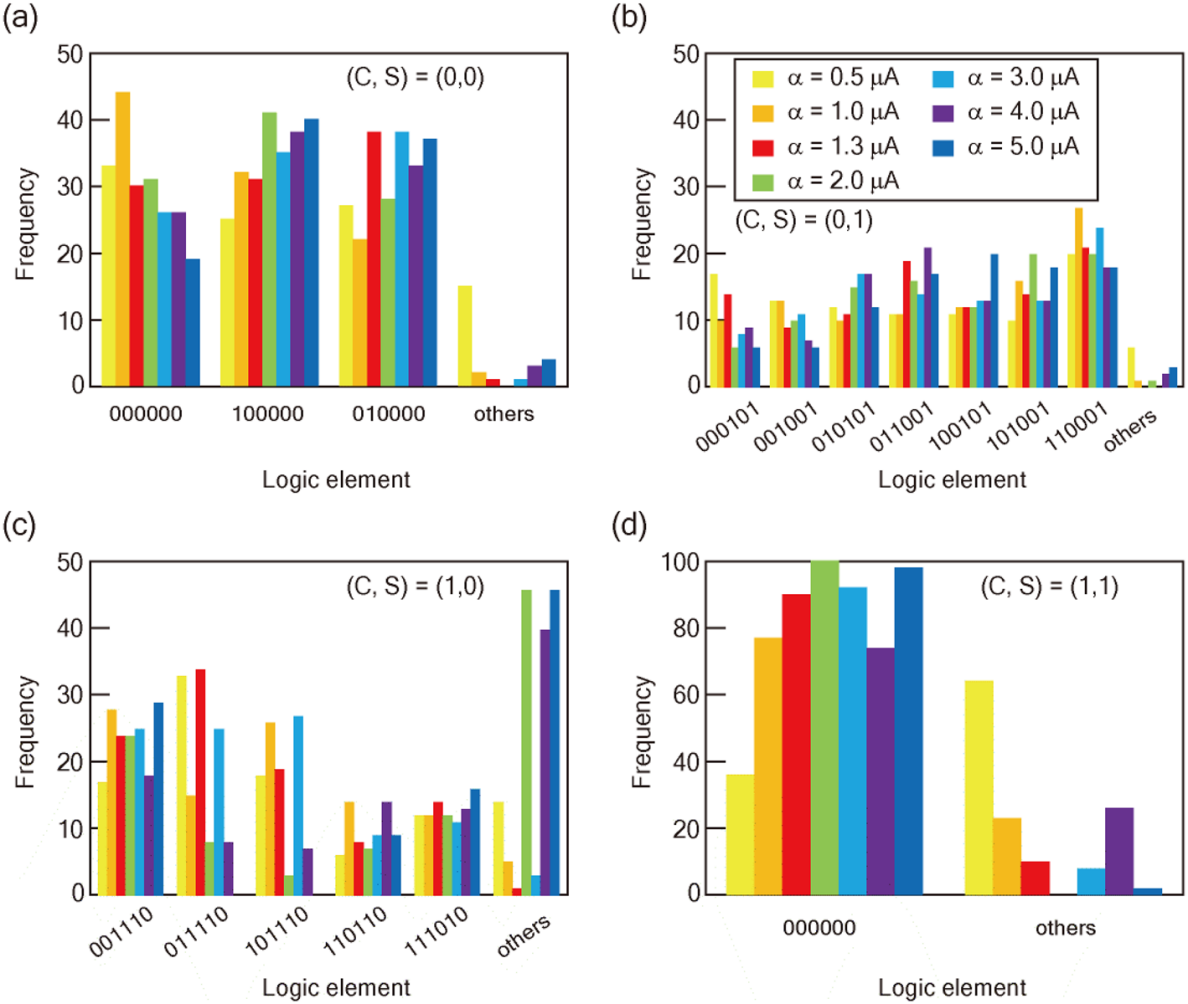
JSIM analysis of the factorization. Frequency distribution of the factorized elements for (**a**) (0,0)_(2)_, (**b**) (0,1)_(2)_, (**c**) (1,0)_(2)_, and (**d**) (1,1)_(2)_. In (1,0)_(2)_, the wrong response increases as α increases; however, every candidate element is still identified.

**Figure 4:**
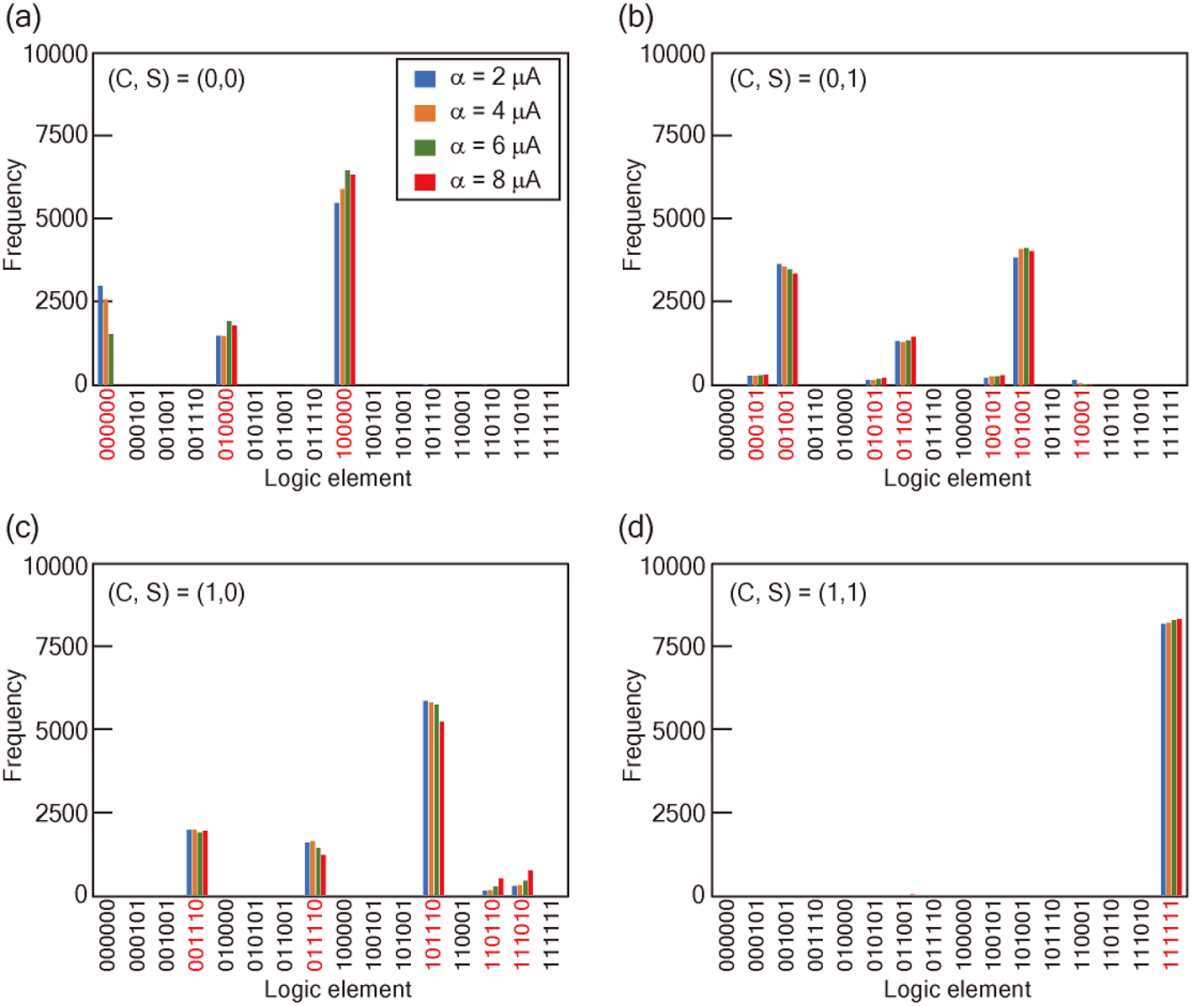
Factorization in experiment. Frequency distribution of factorized elements for (**a**) (0,0)_(2)_, (**b**) (0,1)_(2)_, (**c**) (1,0)_(2)_, and (**d**) (1,1)_(2)_ in MU2 at *T*_a_ of 100 μs. The frequency is modulated by offset current α. We can identify every candidate element for quantum annealing with α of less than 4.

Figure 3 shows JSIM analysis of the α dependence of the factorization in MU2 based on the theoretical degeneracy point. The factorization of (0,0)_(2)_, (0,1)_(2)_, (1,0)_(2)_, and (1,1)_(2)_ represents 3, 7, 5, and 1 types of candidate solution, respectively. Here, we focus on the factorization of (1,0)_(2)_. The frequency in the element (*X*, *Y*, *Z*, *D, C, S*) = (0, 1, 1, 1, 1, 0) and (1, 0, 1, 1, 1, 0) varies dynamically as α increases, and the error response increases with α. These trends correspond to the decrease of the success probability at α > 1.3 in Fig. 2b. The error components consist chiefly of different combinations from the 16 components that minimize the energy in the Hamiltonian. These results indicate that the energy diagram for the factorization of (1,0)_(2)_ is not stable as α increases, but has a sparse distribution of low energy states that correspond to factorization elements. Figure 4 shows the experimental α dependence of the factorization at *T*_a_ = 100 μs. All candidate elements are observed in each factorization, indicating that the response is better than that in MU1 reported in Ref.^24^. The frequency distributions of the factorized components at *T*_a_ = 1 and 15 μs are summarized in Supplementary Fig. S4. There is a different α dependence between *T*_a_ = 1 and 100 μs, probably related to the trend in Supplementary Fig. S2b, which reflects the sparse distribution of low energy states. In the factorization of (0,1)_(2)_, all candidate components are identified with α less than 4. The variation of the components in the factorization is improved in MU2 compared with that in MU1. The response of the component (*X*, *Y*, *Z*, *D, C, S*) = (0, 0, 0, 0, 0, 0) in the factorization of (0,0)_(2)_ indicates that there is an optimum value of α for accurate factorization. Figure 2c–f show the experimental α dependence of the success probability. The success probability reaches a maximum at α of about 2. This α dependence is consistent with the trends discussed so far. In addition, a slight decrease in the success probability after the peak in Fig. 2b is observed.

## Discussion

Here, we discuss why the theoretical and experimental degeneracy points are different. The theoretically estimated degeneracy point is consistent with that evaluated using JSIM, which means that the superconducting quantum circuit shows theoretical behavior if the Hamiltonian is precisely implemented. However, we focus on the difference between the experimental results and the JSIM analysis. There is an offset magnetic flux generated by a surrounding circuit and qubits in experiment. We can identify the effect of the offset flux from the trend in the state-1 probability (see Supplementary Fig. S5). In the experiment, it is necessary to calibrate the effect of the offset flux. However, the calibrated degeneracy point is still different from the theoretical degeneracy point. Here, improving the uniformity of component generation and the variety of factorized components in MU2 compared with those in MU1 may indicate an important design issue. The undesirable local minima of MU1 is suppressed in MU2 (Supplementary Fig. S6). In the fabrication of MU2, heat treatment at 220 °C is applied, which controls *I*_c_; however, we should consider whether treatment causes an irreversible change in the characteristics of the Josephson junctions. The boundary condition of the irreversible change is probably between 220 and 230 °C (Supplementary Fig. S7). Supplementary Fig. S5 shows the state-1 probability of each qubit consisting of MU2. For the state-1 probability in qubit-5 (C), the transition direction is unstable around a probability of 0.5. This behavior is not observed in the same qubit in MU1. The state-1 probability indicates that the irreversible change in the characteristics in the Josephson junctions does not occur uniformly around 220 °C. These results indicate that the characteristics of the Josephson junction were degraded by the thermal treatment in MU2, and this is main reason for the difference between the theoretical and experimental degeneracy points.

It is possible to obtain the intended logic component by using α, even if it is far from the degeneracy point. However, we should take care to select an appropriate value of α. Supplementary Fig. S8 shows the factorizations in MU2 performed using OP1 (where several elements of 16 candidate do not occur as shown in Supplementary Fig. S9b). Factorization based on the theoretical degeneracy point is also investigated (see Supplementary Figs. S10 and S11). The success probabilities were above 80% with α of around 1. The variation of the probability with α is larger in Supplementary Fig. S11 than in Fig. 2c. Although the success probability is high, the components in the factorization are biased. These results indicate that the energy potential has a sparse distribution of global minima. Consistent experimental and theoretical degeneracy points are obtained by the following changes. The first is revising the variation of *L* between qubits. This is mainly due to the design of qubit-5 (C), which has large rings for qubit interactions. The second is decreasing *I*_c_ by reducing the size of the Josephson junctions. Because we mainly use Josephson junctions with sizes of 1–7 μm^2^, the amount of damage from the fabrication process, especially ion-beam etching, is expected to be small. We will control the size of the junction and its *I*_c_ evaluation on the submicrometer squared scale. In future work, the target *I*_c_ in the qubit will be 1–3 μA.

As similarly in the classical multiplier^32^, our proposed method for the prime factorization can be scaled up by adding MUs (see “Concept of scalable factorization circuit” in the “Supplementary Note”). Supplementary Fig. S12 shows JSIM analysis in a case of factorization of “6” with the 4-bit factorization circuit, where the Hamiltonian shown in Fig. 1d is implemented. Two candidates in true combinations of *M* = (*X*_2_ *X*_1_)_(2)_ and *N* = (*Y*_2_ *Y*_1_)_(2)_ are obtained with success probability above 80%. Note that this result corresponds to success probability in the MU solely as shown in Fig. 2c–f. This suggests that a scalable factorization system could be built.

We have fabricated an MU with a success probability above 80% for factorization. The MU is robust for factorization because we can tune the conditions via the offset current. The functionality is easily expandable by adding extra MUs. We believe that these results contribute to conventional computing as well as quantum computing because our approach provides an alternative method for solving prime factorization.

## Methods

### Superconducting flux qubit

The qubits used in this experiment are superconducting compound Josephson junction rf-SQUID flux qubits, which is a similar configuration to that described by Harris et al.^17,18^. We fabricate a QA circuit using a process that creates four Nb layers and a Josephson junction with a critical current density of 1 μA/μm^2^. Though single Josephson junction with size of 0.3 μm^2^ is fabricated, size controllability has not been established. In order to create a stable structure for the qubit, we adopted the Josephson junction with size of 6.25 μm^2^ for the superconducting quantum circuit. In MU2, heat treatment at 220 °C is applied after the fabrication to reduce *I*_c_ of the Josephson junction. Because the qubit is composed of two superconducting loops, consisting of a large loop and an inserted small loop with the Josephson junctions, there are two flux degrees of freedom, which are controlled by eternal flux biases Φ_2_ and Φ_1_. The rf-SQUID has two bistable states with persistent current flowing clockwise or counterclockwise through the large loop when Φ_1_ of Φ_0_/2 (Φ_0_ is the flux quantum) is applied. These two states correspond to logical 1 and 0 states in the qubit. Measurement details are described in “Experimental configuration” of the “Supplementary Methods”.

### Design of the MU

Inductances (*L*) and mutual inductances (*M*) are extracted from the layout of the superconducting quantum circuit using InductEX^36^. In the qubit, a bistable energy state can be achieved by coordinating the value of a dimensionless factor, β_L_ = 2π*LI*_c_/Φ_0_. MU is composed of six superconducting flux qubits, which have all-to-all connectivity. The two types of MU consist of the same superconducting circuits (*L* = 287.2 ± 8.0 pH) with different *I*_c_, depending on whether they are thermally annealed at 220 °C.

### JSIM analysis

The MU circuit model is constructed and analyzed by a JSIM^35^. *L* and *M* parameters extracted from the MU layout are used in the circuit model. Owing to the time constraint, *T*_a_ settled in 1 μs. The noise current, which reproduces the probability of the qubit state transition in the 10 mK experiment at *T*_a_ = 100 μs, is used. Each multiplication and factorization performed by individual α is performed with 100 iterations.

## Supplementary Information

Supplementary Information.
