# Supplementary material for: Factorization by quantum annealing using superconducting flux qubits implementing a multiplier Hamiltonian
Source: Sci Rep. 2022 Aug 11;12:13669. doi: 10.1038/s41598-022-17867-9 (PMC9372081; doi:10.1038/s41598-022-17867-9)
Supplement: Supplementary file 1 — Supplementary Information. [file 41598_2022_17867_MOESM1_ESM.docx]

Supplementary Materials for

**Factorization by Quantum Annealing Using Superconducting Flux Qubits Implementing a Multiplier Hamiltonian**

Daisuke Saida^1*^, Mutsuo Hidaka^1^, Kentaro Imafuku^1^ and Yuki Yamanashi^2^

^1^National Institute of Advanced Industrial Science and Technology

^2^Yokohama National University

*Corresponding author: saida.daisuke@aist.go.jp

**Supplementary Methods**

Here, we discuss the elicitation process for the theoretical degeneracy point. As a general example of the quantum annealing, we consider the quantum dynamics governed by the Hamiltonian

$H=\sum_{i} h_{i}\sigma_{z}^{(i)}+\sum_{i>j} J_{ij}\sigma_{z}^{(i)}\sigma_{z}^{(j)}$ , (1)

where *σ_z_*^(i)^ is *z* Pauli matrix acting on qubit *i*^1-3^. In the multiplier unit (MU) with the problem Hamiltonian^4-6^ shown in Fig. 1(b), the dimensionless local biases of *h_i_* and pairwise couplings *J_ij_* take the values

$h_{1}:h_{2}:h_{3}:h_{4}:h_{5}:h_{6}:J_{12}:J_{13}:J_{14}:J_{15}:J_{16}:J_{23}:J_{24}:J_{25}:J_{26}:J_{34}:J_{35}:J_{36}:J_{45}:J_{46}:J_{56}=-1:-1:-2:-2:+4:+2:+1:+2:+2-4:-2:+2:+2:-4:-2:+4:-8:-4:-8:-4:+8$. (2)

Here, *h_i_* and *J_ij_* are given by

$h_{i}=M_{i}\cdot I_{hi}\cdot I_{qi}$,

$J_{ij}=M_{ij}\cdot I_{qi}\cdot I_{qj}$, (3)

where *M_i_* is the mutual inductance between qubit *i* and the local bias line, *I_hi_* is the current flowing through the local bias line, *I_qi_* is the persistent current in qubit *i*, and *M_ij_* is the mutual inductance between qubits *i* and *j*, respectively. Due to the symmetry between $\sigma_{z}^{(1)}$ and $\sigma_{z}^{(2)}$, the dimensionless local biases of *h_i_* and pairwise couplings *J_ij_* are

$h_{1}=h_{2}, J_{13}=J_{23}, J_{14}=J_{24}, J_{15}=J_{25}, J_{16}=J_{26}$. (4)

Similarly,

$h_{3}=h_{4}, J_{13}=J_{14}, J_{23}=J_{24}, J_{35}=J_{45}, J_{36}=J_{46}$. (5)

Consequently, the first requirement of an implementation of the MU can be written as

$M_{1}=M_{2}, M_{13}=M_{23}, M_{14}=M_{24}, M_{15}=M_{25}, M_{16}=M_{26}, {M_{3}=M_{4},M}_{13}=M_{14}, M_{23}=M_{24}, M_{35}=M_{45}, M_{36}=M_{46}$.

(6)

The second requirement of the implementation of the MU is given by

$\frac{M_{13}}{M_{12}}=\frac{M_{34}}{M_{13}}=\frac{M_{35}}{M_{15}}=\frac{M_{36}}{M_{16}}$,

$\frac{M_{56}}{M_{15}}=\frac{M_{16}}{M_{12}}$. (7)

We should satisfy these requirements for direct implementation of the MU Hamiltonian in the superconducting quantum circuit. Supplementary Tables S1 and S2 show the constraints of the Hamiltonian implementations and the implemented relationships in the circuit. The mutual inductances used in Supplementary Eqs. (6) and (7) are extracted from the layout of the MU by InductEX. We confirmed that the inductances extracted by InductEX agreed with experimental values measured at the MU. The design of the MU generally satisfies two requirements.

Using Supplementary Eqs. (2) and (3), the current relationship at the degeneracy point of the MU can be derived.

$I_{h2}=I_{h1}$,

$I_{h4}=I_{h3}=\frac{M_{1}}{M_{3}}\cdot\frac{M_{13}}{M_{12}}\cdot I_{h1}$,

$I_{h5}=\frac{M_{1}}{M_{5}}\cdot\frac{M_{15}}{M_{12}}\cdot I_{h1}$,

$I_{h6}=\frac{M_{1}}{M_{6}}\cdot\frac{M_{16}}{M_{12}}\cdot I_{h1}$,

$I_{q1}=-\frac{M_{1}}{M_{12}}\cdot I_{h1}$,

$I_{q2}=I_{q1}$,

$I_{q3}=-2\frac{M_{1}}{M_{13}}\cdot I_{h1}$,

$I_{q5}=4\cdot\frac{M_{1}}{M_{15}}\cdot I_{h1}$,

$I_{q6}=2\cdot\frac{M_{1}}{M_{16}}\cdot I_{h1}$, (8)

where *I*_q_*_i_* (*i* = 1–6) is the persistent current in qubit *i*.

**Experimental configuration.** Each qubit state is evaluated by applying a time-dependent transverse field to the quantum annealing. The annealing effect is controlled by the annealing time (*T*_a_) of the field. Supplementary Fig. S1(a) shows the experimental setup in each qubit, consisting of the superconducting quantum circuit. Arbitrary wave generators are used to apply flux with an accurate time schedule. *I*_trans_ is the current for the transverse field, *I*_QFP_ is the flux-injection current for a quantum flux parametron (QFP), *I*_bias_sq_ is the drive current for a dc superconducting quantum interference device (SQUID), *I*_flux_sq_ is the modulation current for flux detection in the dc-SQUID, and *I*_h_ is the external bias current in the qubit. The annealing schedule is controlled by the rise time in *I*_trans_. The maximum amplitude of *I*_trans_ corresponds to injection of the quantum flux, Φ_0_ (2.07 × 10^−15^ Wb), to the rf-SQUID in the qubit. Here, Φ_1_ and Φ_2_ described in Methods are flux induced by *I*_trans_ and *I*_h_, respectively. After the Φ_0_ injection, the QFP and the dc-SQUID are activated. The QFP detects flux due to the persistent current in the qubit and transfers it to the dc-SQUID with amplification. The dc-SQUID is tuned by applying an external flux to respond to clockwise current in the qubit. Supplementary Fig. S1(b) shows typical waveforms monitored by an oscilloscope for each current. The qubit state is detected in 0.6–0.8 ms and is typically evaluated over 10^4^ iterations. We search combinations of currents (*I*_h_*_i_*, *i* = 1–6 in the MU), which provides every logic component during 10^4^ iterations.

The superconducting quantum circuit is mounted in a dilution refrigerator and is cooled at 10 mK. The thermal energy at 10 mK is estimated to be 1.4 × 10^-25^ J. The energy in the potential of the rf-SQUID in the qubit around its bottom is about 2.0 × 10^-21^ J. Disturbance due to the thermal energy is neglectable in our experiment. We consider the effect of flux trapping inside and around the circuit. If flux trapping occurs, the state transition of the qubit is directly affected. In this case, the state-1 probability mainly shows saturation in the middle of the state transition. To suppress flux trapping, we use a magnetic shield that surrounds the circuit. In addition, the state-1 probability of each qubit in the MU is evaluated prior to the main experiment. Thus, we prevent flux trapping. Suppression of electric noise is also vital. To evaluate the effect of electric noise, we measure the critical current (*I*_c_) in the single Josephson junction with size of less than 1.0 μm^2^. Supplementary Fig. S1(c) shows the measured and designed values of *I*_c_. Data represented in this figure is one of the best performance. Size controllability of the Josephson junction in process to process has not been established. The Josephson junction is circular in this experiment. *I*_c_ has a quadratic dependence on the diameter of the Josephson junction. *I*_c_ of 0.28 μA is clearly identified, indicating that the electric noise is suppressed. The noise level of the instrument is also a source of electric noise, and thus instrument selection for *I*_h_ is crucial. Supplementary Fig. S1(d) shows the *I*_h_ dependence of the state-1 probability using a current source with a noise floor on order of pA/(Hz)^0.5^. We cannot distinguish *T*_a_ dependence. For expedience, we define the region with the state-1 probability of 0.1–0.9 as a gray zone. The gray zone is about 0.3 μA at *T*_a_ = 100 μs, which is larger than that in Supplementary Fig. S1(e), which is evaluated using current source with a noise floor on order of fA/(Hz)^0.5^. Thus, we use the current source with a noise floor on order of fA/(Hz)^0.5^ for *I*_h_. We focus on the value of *I*_h_ where the state-1 probability is 0.5. *I*_h_ rapidly decreases between 20 μs and 1 ms and is saturated above 1 ms. This acceleration, indicating a rapid state transition, is probably related to quantum mechanical tunneling. The experimental features of superconducting flux qubits agreed well with the results of quantum mechanical simulations in a D-wave system with fewer than 8 qubits^7^. Because the energy scale of our qubit is similar to that used in Ref. 7, the quantum effect, shown in the simulation performed using D-wave systems, also appears in our experiment because of the noise suppression.

**Supplementary Note**

**Detailed characteristics of the MU.** Here, we present all the results for the 16 multiplications and four factorizations. First, we investigate the external current *I*_h_ combinations by which the sixteen candidate components are generated most frequently. Supplementary Fig. S2 shows the histograms for MU1 and MU2 at currents where all 16 components are observed experimentally with 10^4^ iterations. (*I*_h1_, *I*_h2_, *I*_h3_, *I*_h4_, *I*_h5_, *I*_h6_) of MU1 and MU2 are (-0.28, -0.23, -0.45, -0.6, 0.37, 0.42) and (-0.3, -0.3, -0.4, -0.4, 0.5, 0.4) [μA], respectively. The frequency of occurrence of each component in MU2 is more uniform than that in MU1. Although the theoretical and experimental degeneracy points are different, we can use these current conditions to generate logic components in the multiplication and the factorization by applying α. Supplementary Fig. S3(a)–S3(p) show histograms of the multiplication in MU2 by applying α to the degeneracy point of (*I*_h1_, *I*_h2_, *I*_h3_, *I*_h4_, *I*_h5_, *I*_h6_) = (-0.3, -0.3, -0.4, -0.4, 0.5, 0.4) [μA] (OP2). α is set between 2 and 8. Histograms are evaluated with *T*_a_ = 1, 15, and 100 μs. In both *T*_a_, all 16 candidate logic components, which are at the minimum energy level in Fig. 1(b), are observed. Multiplication of (*X*, *Y*, *Z*, *D*) = (1, 1, 1, 1) and (1, 1, 1, 0) indicate that large values of α are not appropriate.

Supplementary Fig. S4 shows offset current α dependencies of success probability for the factorization in MU2 with *T*_a_ of 1, 15, and 100 μs. Although total success probability in each factorization is small, the variation of the generated component is better for *T*_a_ = 1 μs than that for *T*_a_ = 100 μs. This may be because the 16 combinations of global minima in the Hamiltonian do not take the same value in MU2. As mentioned in “Effect of thermal treatment” below, thermal treatment may change the characteristics of the qubits in MU2 slightly. This would change the value of the energy in the 16 combinations, and thus the qubit state is trapped at the bottom of the energy that first appears at *T*_a_ = 1 μs. In contrast, at *T*_a_ = 100 μs, the qubit state relaxes to a lower energy, which is unintentionally biased.

**State transition in each qubit.** Here, we focus on the characteristics of each qubit in the MU. Supplementary Fig. S5 shows the local bias current dependence of the state-1 probability in each qubit in MU2 at *T*_a_ = 100 μs. In our device structure, to reduce the overhead of the current sources, the current path of *I*_trans_ (similar to *I*_QFP_ and *I*_bias_flux_) is common between qubits. Even in the measurement of a single qubit, flux from the surrounding circuit affects the state-1 probability, as observed in the offset current. From this measurement, we can evaluate the offset current, which is related to the offset field generated from the surrounding circuit during the operation in the MU. The offset currents measured in qubit-1 to qubit-6 are (-0.07, -0.23, -0.09, -0.42, -0.70, 0.00) [μA], respectively.

Focusing on the state-1 probability in qubit-5 (C), the transition direction is unstable around a probability of 0.5. This behavior is reproduced in measurements at several times and is not observed in the corresponding qubit in MU1. The state-1 probability indicates that the irreversible change in the characteristics in the Josephson junctions occurs in qubit-5 (C). In addition, the state-1 probability in each qubit suggests that the irreversible change does not occur uniformly with the thermal treatment at 220 °C.

**Energy potential.** The shape of the energy potential is related to the state transition in the qubit during the quantum annealing process. Supplementary Fig. S6 shows the numerical calculations of the energy potentials in the qubit^8^. In MU1, there is an undesirable local minimum (red dashed circle). Suppression of the local minimum in the potential shape contributes to the accurate operation of the MU. The shape of the energy potential is better in MU2, in which *I*_c_ is lower. The dimensionless factor, *β*_L_ = 2π*LI*_c_/*Φ*_0_, is 10.9 and 6.5 in MU1 and MU2, respectively. In the MU2, the barrier height between two global minima is reduced, which may help to increase the ratio in the quantum mechanical tunneling. In future work, we will use *I*_c_ values of 1–3 μA in the MU. This would decrease *β*_L_ to 1.7–5.2, allowing sufficient accuracy to check the consistency between theoretical and experimental degeneracy points.

**Effect of thermal treatment.** We discuss the post-processing, which can control *I*_c_ in the qubit. Supplementary Fig. S7 shows modulation of the *I*_c_ by the thermal treatment. *I*_c_ of the Josephson junction (square shape) with width of 2, 3, and 4 μm is investigated. Josephson junctions with size of 6.25 μm^2^ are used in MU1 and MU2. Thermal treatment decreases *I*_c_, and the rate of the decrease with the temperature changes above 220 °C. The thermal treatment at 230 °C decreases *I*_c_ and the signal ratio (corresponding to reduction of the signal-to-noise ratio) in the measurements. These results indicate we can control the value of *I*_c_ by up to 60% after device fabrication with thermal treatment at 220 °C.

As shown in Supplementary Fig. S5(e), unstable state transition occurs after the thermal treatment. To improve *I*_c_ below 3.75 μA, reduction of the size of the Josephson junction is more appropriate. We plan to improve the process of the superconducting quantum circuit composed of the Josephson junction with size of 1-3 μm^2^ (corresponding to *I*_c_ of 1-3 μA) in our future work.

**Impact of use in the degeneracy point.** Here, we focus on obtaining the intended logic element by using the offset current even under conditions different from the degeneracy point. Three types of degeneracy point are considered. The first type is the experimentally obtained current condition of (*I*_h1_, *I*_h2_, *I*_h3_, *I*_h4_, *I*_h5_, *I*_h6_) = (-0.28, -0.23, -0.45, -0.60, 0.37, 0.42) [μA] in MU1 (OP1). Later, we apply OP1 to the factorization of MU2. The second type is the experimentally obtained current condition of (*I*_h1_, *I*_h2_, *I*_h3_, *I*_h4_, *I*_h5_, *I*_h6_) = (-0.30, -0.30, -0.40, -0.40, 0.50, 0.40) [μA] in MU2 (OP2). The results of the factorization at OP2 are shown in Fig. 2(c)–2(f) and 4. The third type is the theoretically evaluated current condition of (*I*_h1_, *I*_h2_, *I*_h3_, *I*_h4_, *I*_h5_, *I*_h6_) = (-0.30, -0.30, -0.60, -0.60, 1.20, 0.60) [μA] (OP3). Supplementary Fig. S9(a)–S9(c) show the histograms with 10^4^ iterations using MU2 with OP2, OP1, and OP3 current conditions, respectively. For OP2, all 16 candidate components occur, whereas for OP1 and OP3, several components do not occur. Supplementary Fig. S8(a)–S8(d) show the factorization of (0,0)_(2)_, (0,1)_(2)_, (1,0)_(2)_, and (1,1)_(2)_ in the experiment at OP1 in MU2, respectively. The offset current and *T*_a_ dependences are investigated. Although some components are not obtained experimentally at *T*_a_ = 100 and 15 μs, all candidate logic components are identified at *T*_a_ = 1 μs. Supplementary Fig. S11(a)–S11(d) show the α dependence of the success probabilities. Compared with the results in Fig. 2(c)–2(f), the success probability depends strongly on α. The success probability reaches a maximum around α of 1.5, and then decreases as α increases further. The success probability trend in Fig. 2(b), in which the probability decreases after the peak value, is also observed in Supplementary Fig. S11(a)–S11(d). Supplementary Fig. S10(a)–S10(d) show the factorization of (0,0)_(2)_, (0,1)_(2)_, (1,0)_(2)_, and (1,1)_(2)_ for OP3, respectively, and some components do not occur. The results show that the factorization components can be obtained using the current conditions away from the degeneracy point. However, we should consider whether the components in the factorization are biased. In addition, *T*_a_ also affects components generated in the factorization. These results indicate that the energy potential has a sparse distribution of global minima.

**Concept of scalable factorization circuit.** In the quantum annealing, a factorization circuit is possibly formed by the multipliers which multiplies the input *M* and *N*. If we fix the output of the circuit to the known bit string *P*, the circuit provides combinations of *M* and *N* after the annealing^4^. The element of the multiplier; named as multiplier unit (MU) in our case, is built from AND gates and full adders^4,9^. Using the ground state spin logic^10^, the MU can be expressed as Hamiltonian shown in Fig. 1(b). The Hamiltonian expression of this idea is proposed in Ref. 5. We have proposed the MU with native implementation of the Hamiltonian in a superconducting quantum circuit^6^.

The superconducting quantum circuit for the prime factorization could be extended easily by increasing the number of MUs. Figure 1(d) shows description of the Hamiltonian in the 4-bit multiplier. Four qubits of C_11_, S_11_, S_10_, S_00_ are components of the product *P* in the multiplications (green circles shown in Fig. 1(d)). Transportations of carries in the classical multiplier corresponds to interaction between qubits in the superconducting quantum circuit. In addition, X_00_ and X_10_ (X_01_ and X_11_, Y_00_ and Y_01_, Y_10_ and Y_11_) are connected to align the input relationship in the classical circuit. We can select input *M* from combinations of X_01_ & X_00_, X_01_ & X_10_, X_11_ & X_00_, X_11_ & X_10_, X_11_ & X_00_ (expected to be the same result). In Fig. 1(d), pairs of two qubits of X_01_ & X_00_ and Y_11_ & Y_01_ are selected as the inputs.

With embedding the Hamiltonian of Fig. 1(d) to the superconducting quantum circuit, 4-bit factorization can be analyzed. Figure S12 shows JSIM analysis in a case of factorization of “6” with the 4-bit factorization circuit. Here, true combinations of *M* = (*X*_2_ *X*_1_)_(2)_ and *N* = (*Y*_2_ *Y*_1_)_(2)_ in the factorization of “6” are two candidates; (*X*_2_ *X*_1_) = (1,0)_(2)_ and (*Y*_2_ *Y*_1_) = (1,1)_(2)_, or (*X*_2_ *X*_1_) = (1,1)_(2)_ and (*Y*_2_ *Y*_1_) = (1,0)_(2)_. These results are obtained above 80 % in iterations (corresponding to success probability in the MU solely as shown in Fig. 2(c)-(f)) Thus, we consider the functionality of our proposed method has expandability by adding extra MUs, suggesting that a scalable factorization system could be built.


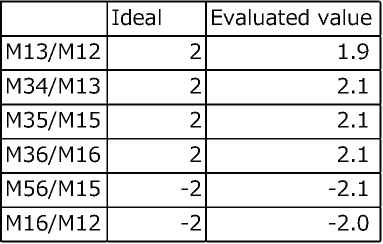


Supplementary Table S2 | Second requirement for direct implementation of the MU Hamiltonian


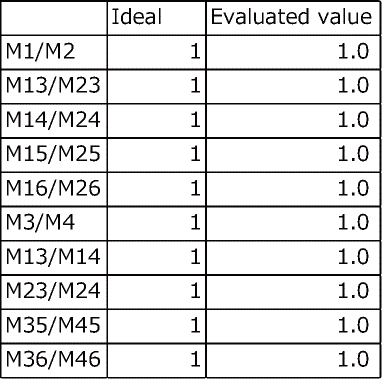


Supplementary Table S1 | First requirement for direct implementation of the MU Hamiltonian


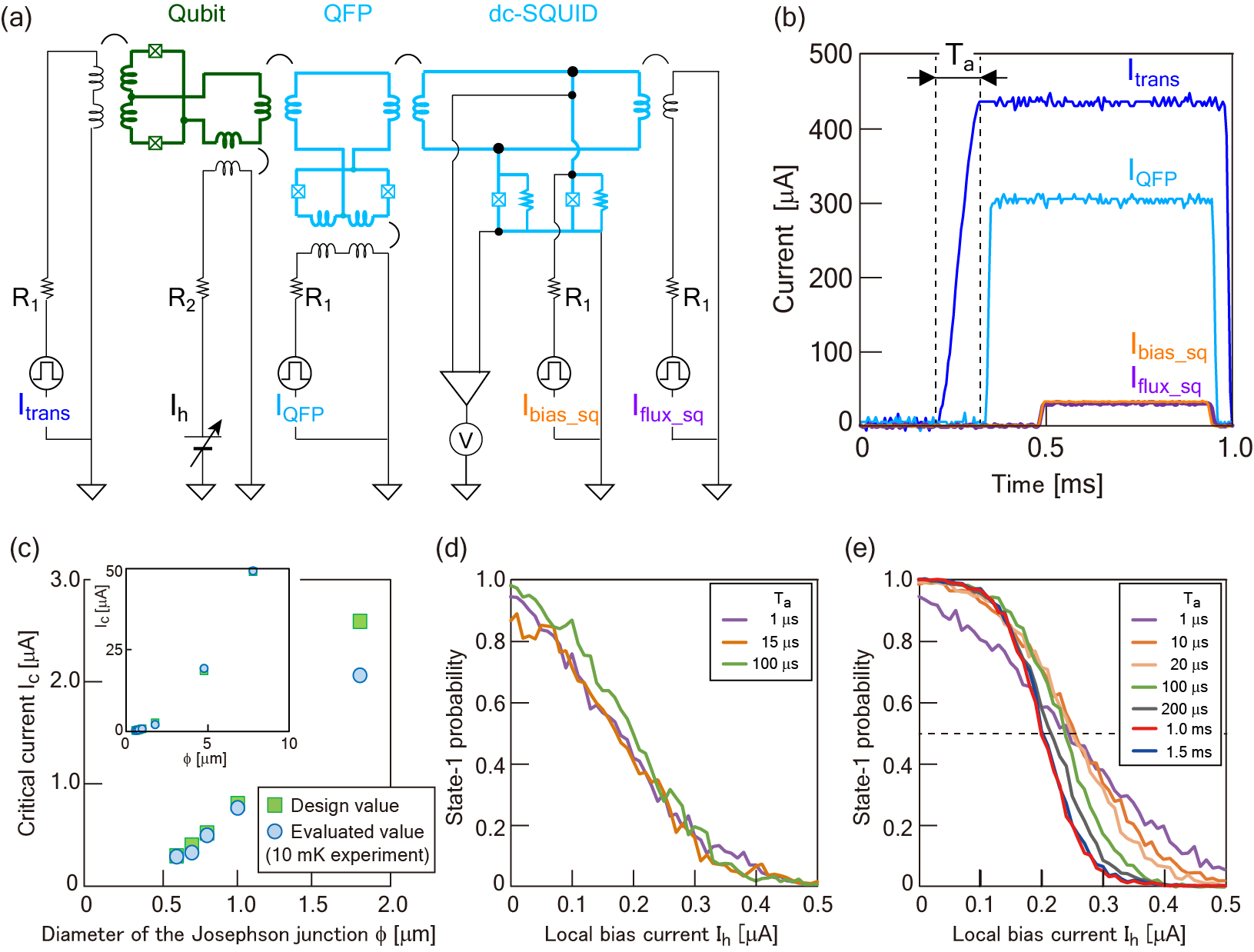


Supplementary Figure S1 | (a) Experimental setup for an individual qubit of the superconducting quantum circuit. The circuit is composed of the qubit (green line) and the readout (blue line). *R*_1_ and *R*_2_ are 10 kΩ and 1 MΩ, respectively. (b) Schedule of applied currents in each qubit. Waveforms are signals observed with an oscilloscope. (c) Measured and designed values of the critical current *I*_c_. These results indicate that the performance of the experimental setup has overcomes the electric noise for current detection above 0.28 μA. State-1 probabilities evaluated using current sources with noise floors of (d) pA/(Hz)^0.5^ and (e) fA/(Hz)^0.5^. In (e), we can identify the annealing time dependence.


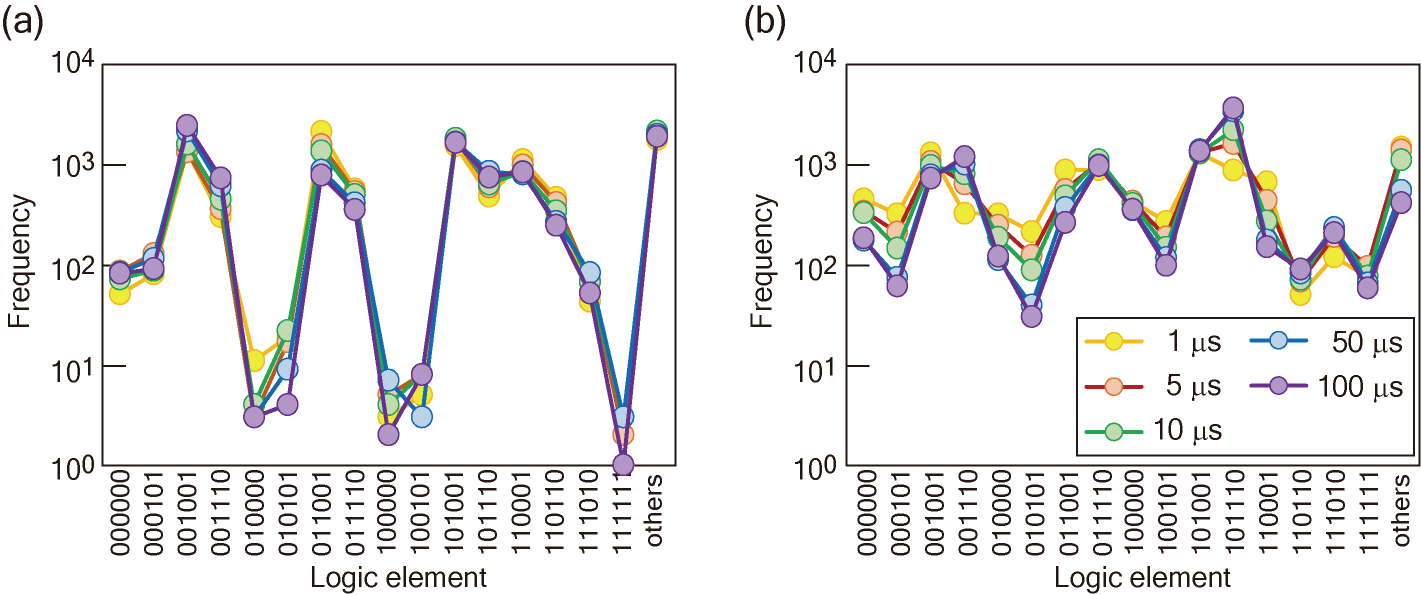


Supplementary Figure S2 | (a) Frequency distribution of each logic component in the experiments at 10^4^ iterations using (a) MU1 (*I*_c_ = 6.25 μA) and (b) MU2 (*I*_c_ = 3.75 μA). *T*_a_ is modulated between 1 and 100 μs. All 16 components are observed in both experiments. The frequency of occurrence of each element in MU2 is more uniform than that in MU1.


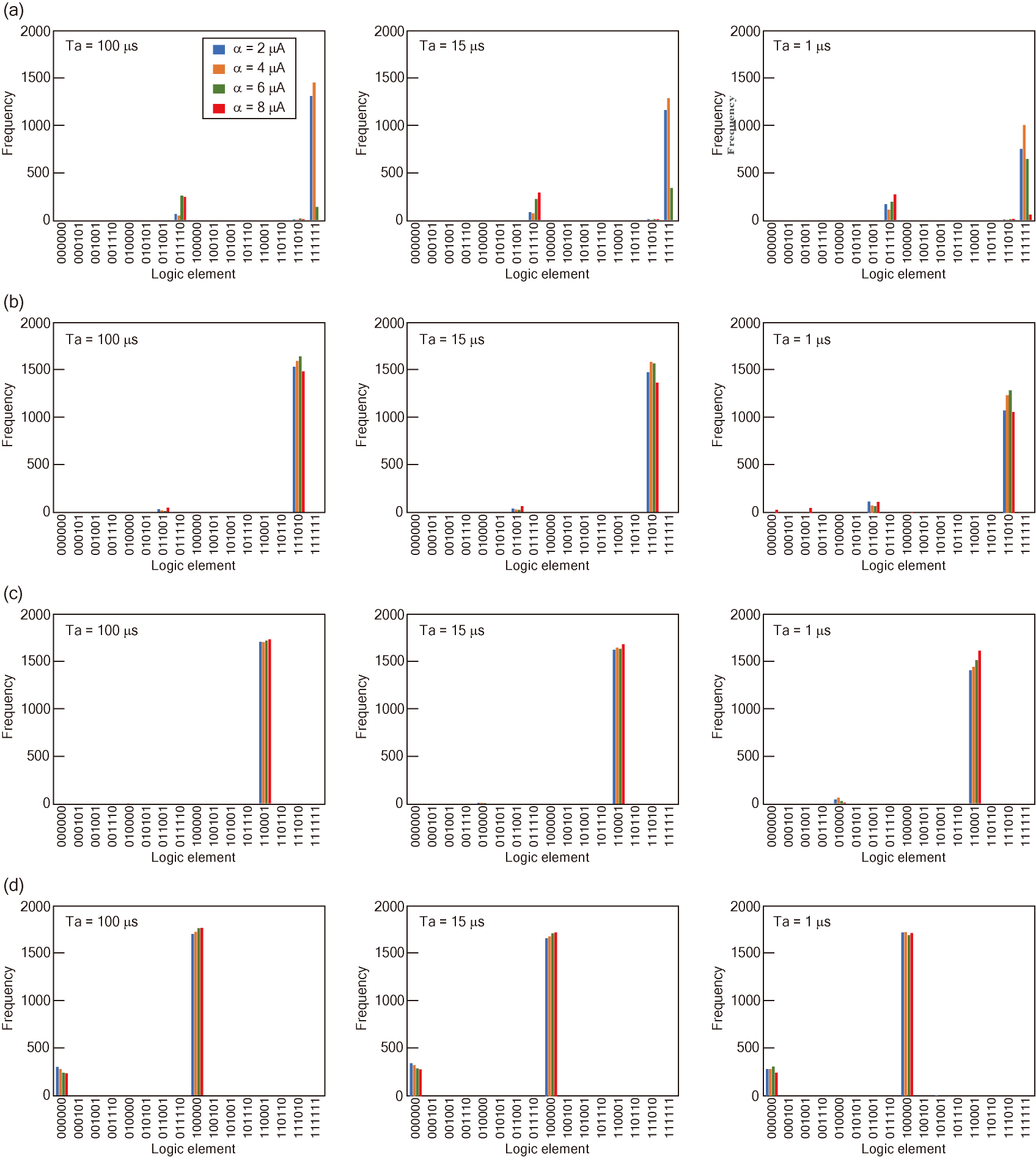


Supplementary Figure S3 | Experimental demonstration of the multiplication of (*X*, *Y*, *Z*, *D*) of (a) (1,1,1,1), (b) (1,1,1,0), (c) (1,1,0,0), and (d) (1,0,0,0).


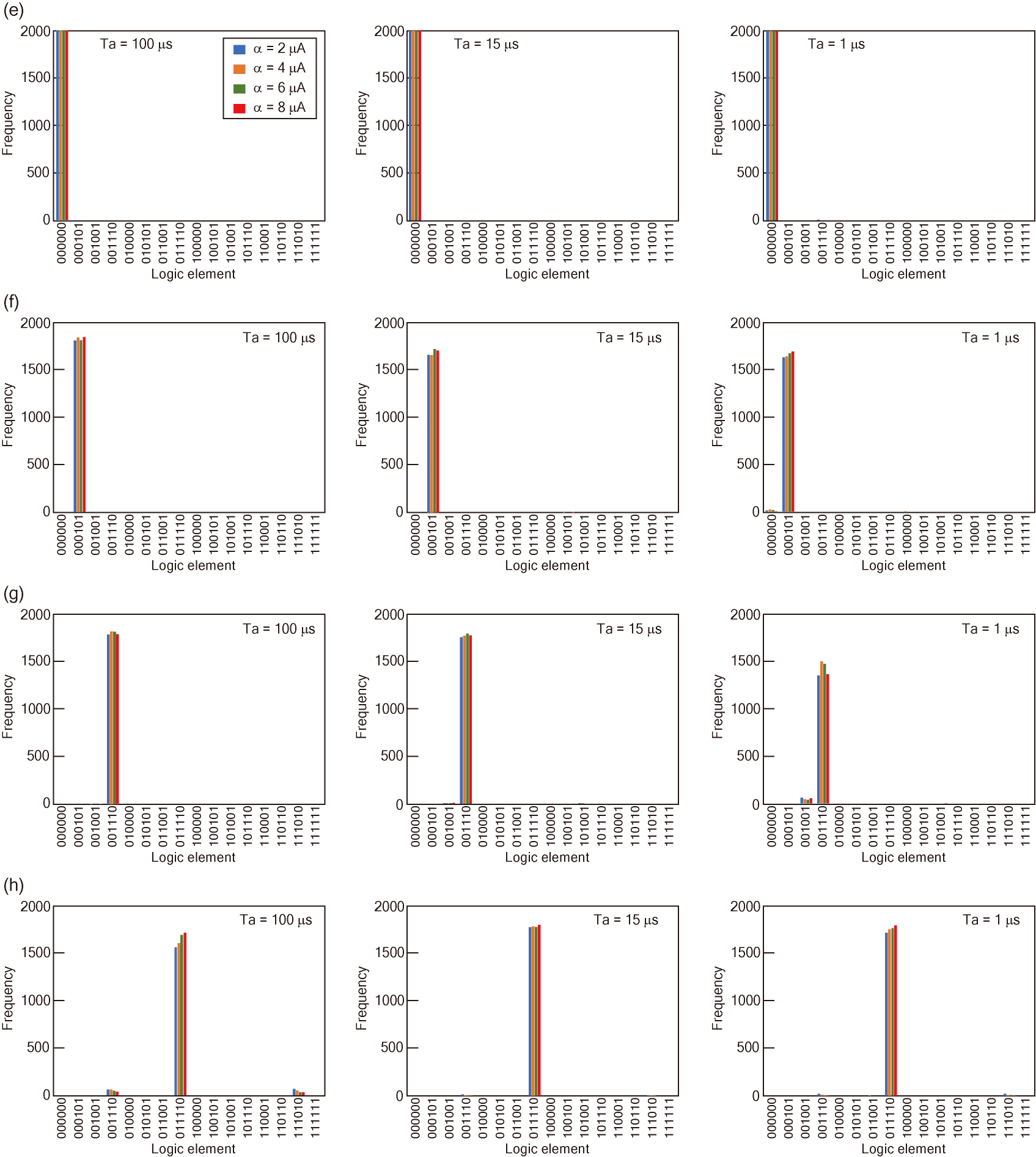


Supplementary Figure S3 | Experimental demonstration of the multiplication of (*X*, *Y*, *Z*, *D*) of (e) (0,0,0,0), (f) (0,0,0,1), (g) (0,0,1,1), and (h) (0,1,1,1).


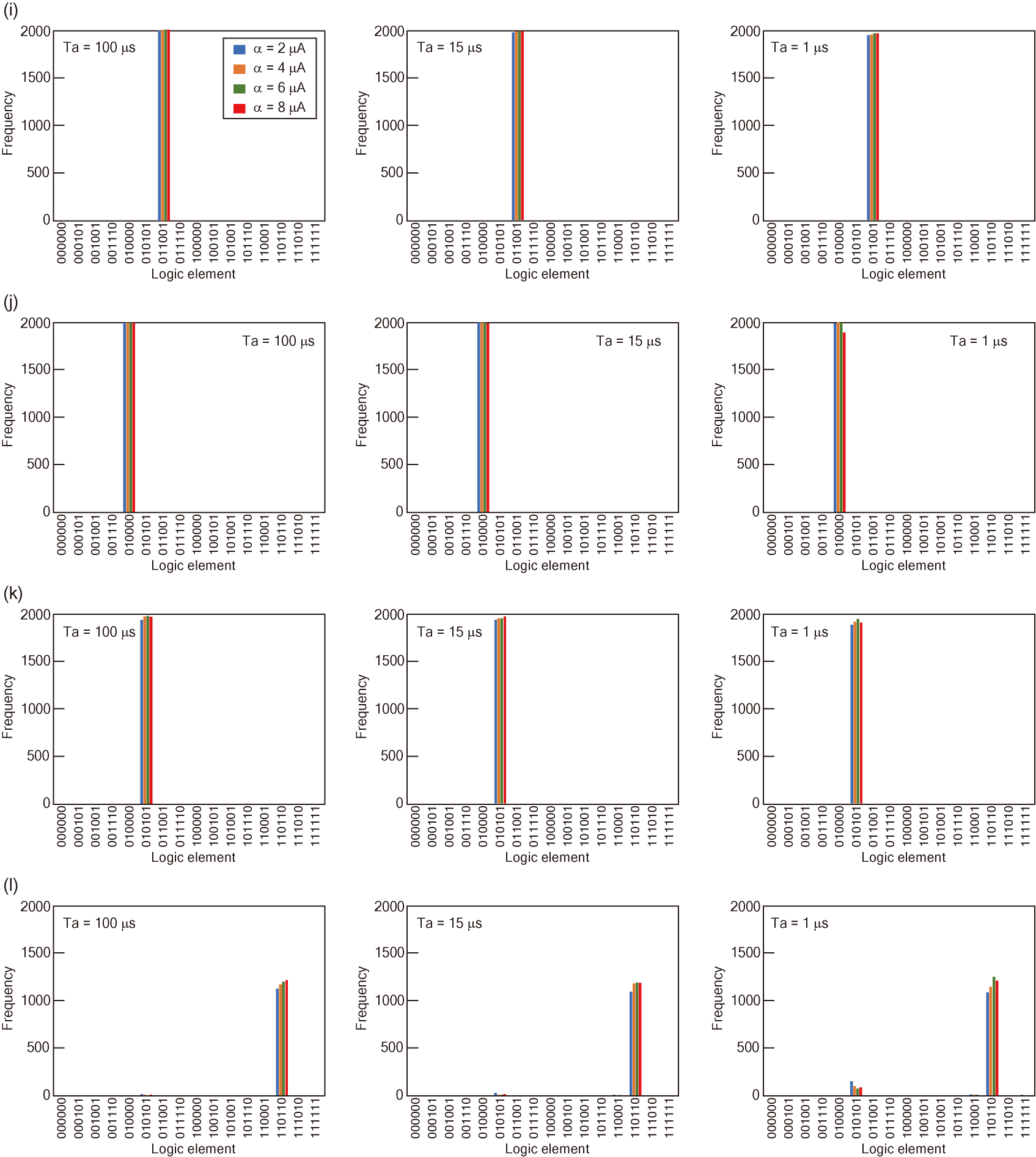


Supplementary Figure S3 | Experimental demonstration of the multiplication of (*X*, *Y*, *Z*, *D*) of (i) (0,1,1,0), (j) (0,1,0,0), (k) (0,1,0,1), and (l) (1,1,0,1).


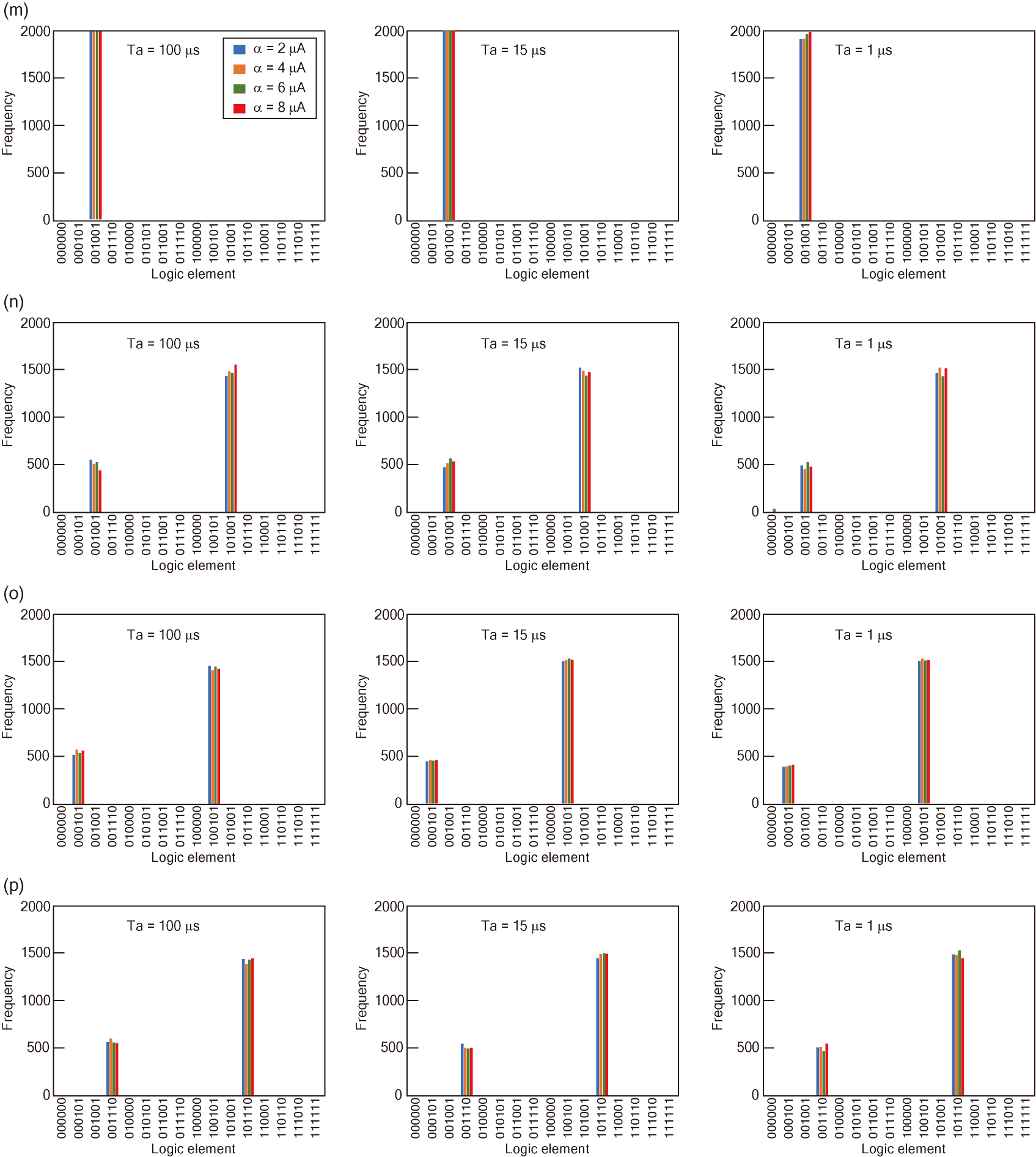


Supplementary Figure S3 | Experimental demonstration of the multiplication of (*X*, *Y*, *Z*, *D*) of (m) (0,0,1,0), (n) (1,0,1,0), (o) (1,0,0,1), and (p) (1,0,1,1).


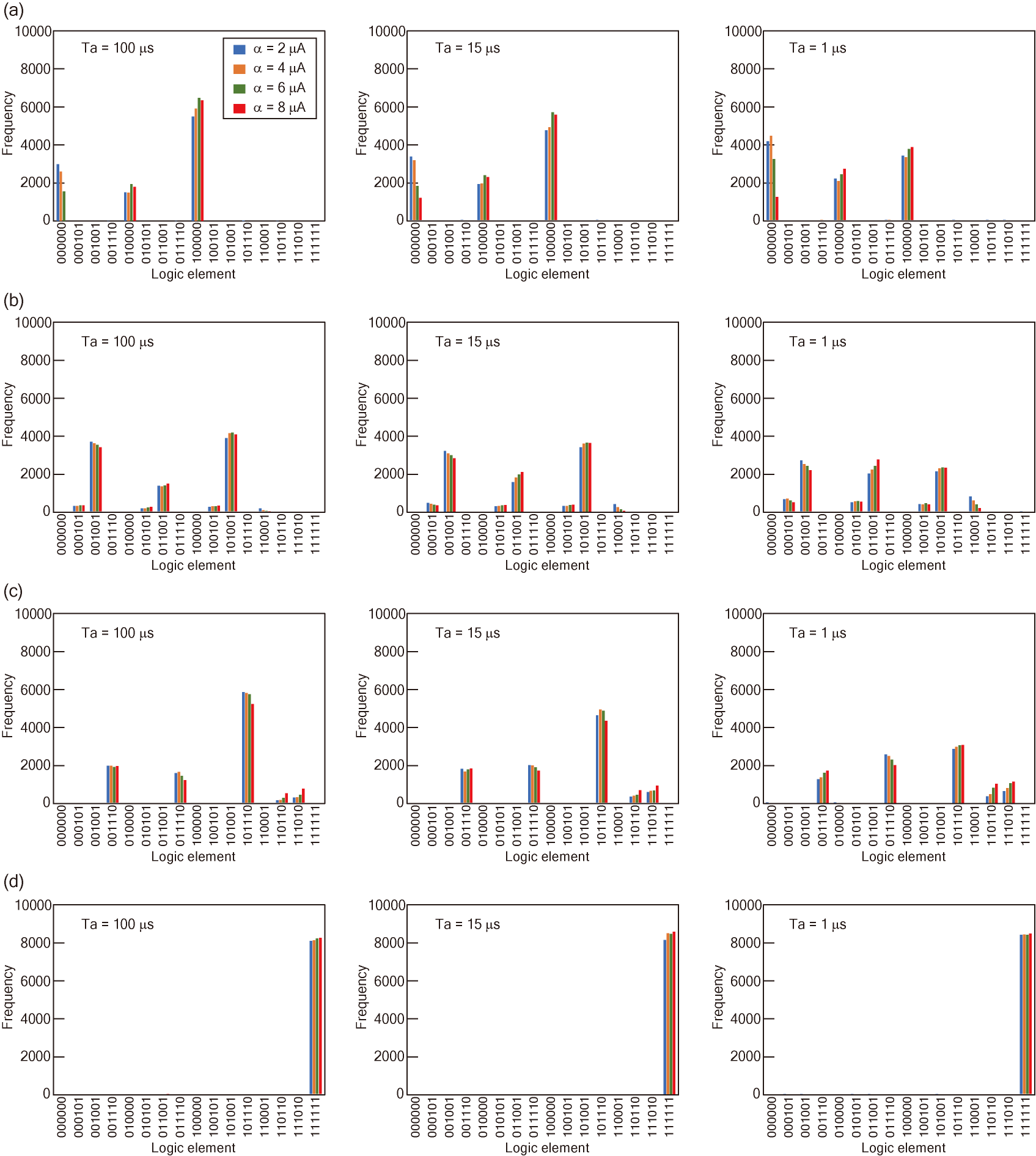


Supplementary Figure S4 | Experimental demonstration of the factorization based on the current condition of OP2 of (*C*, *S*) of (a) (0,0), (b) (0,1), (c) (1,0), and (d) (1,1).


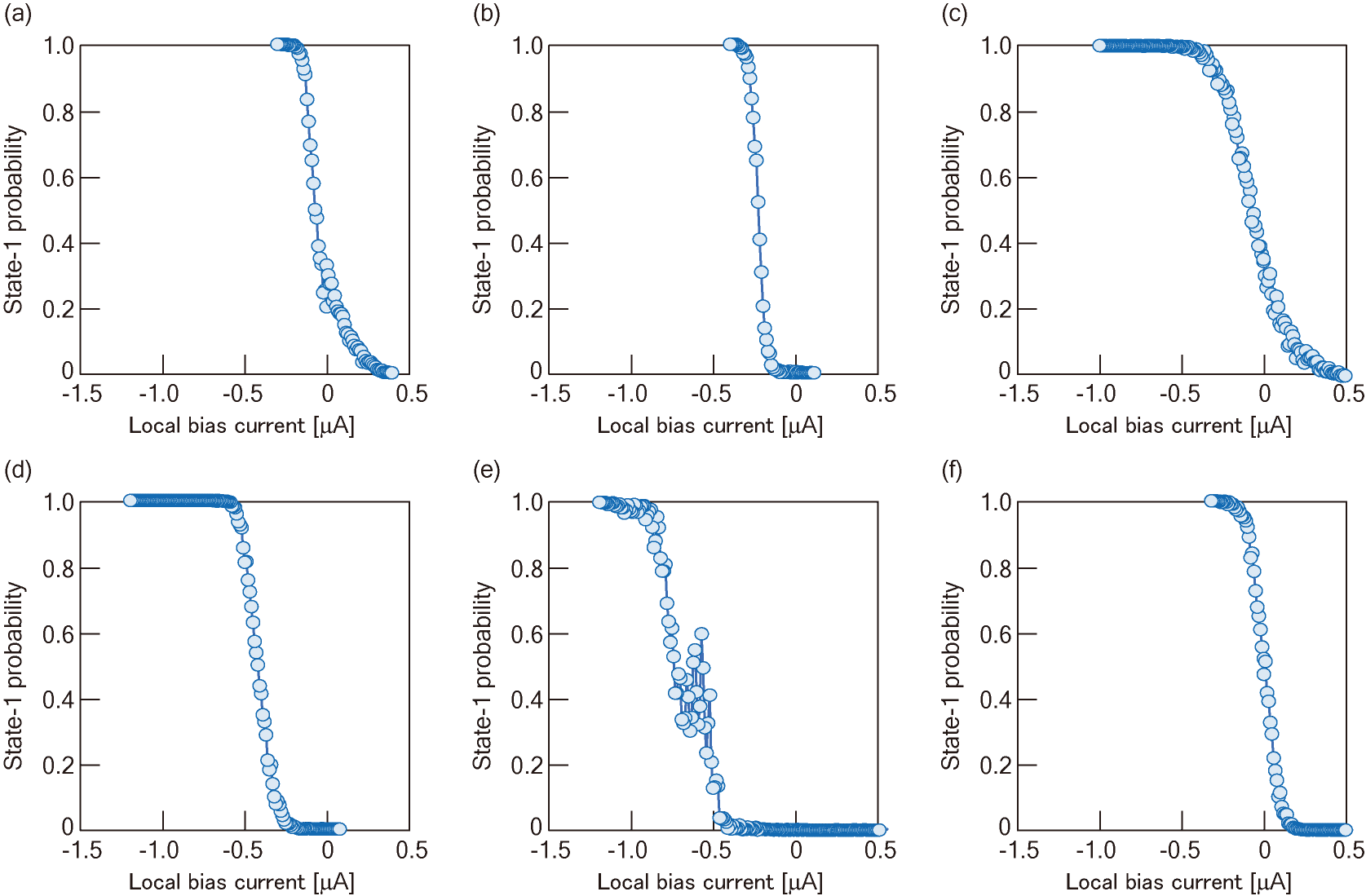


Supplementary Figure S5 | State-1 probabilities of (a) qubit-1 (*X*), (b) qubit-2 (*Y*), (c) qubit-3 (*Z*), (d) qubit-4 (*D*), (e) qubit-5 (*C*), and (f) qubit-6 (*S*) at *T*_a_ = 100 μs in MU2. Each plot is evaluated with 10^3^ iterations.


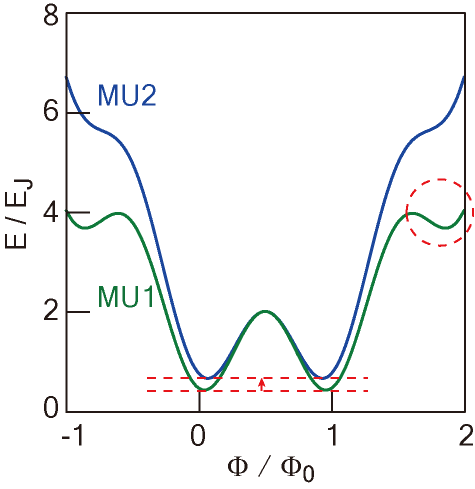


Supplementary Figure S6 | Energy potential of rf-SQUIDs in MU1(*I*_c_ = 6.25 μA) and MU2(*I*_c_ = 3.75 μA). The horizontal axis corresponds to the quantum flux applied through current *I*_h_. The undesirable local minimum in MU1 is indicated by the red dashed circle.


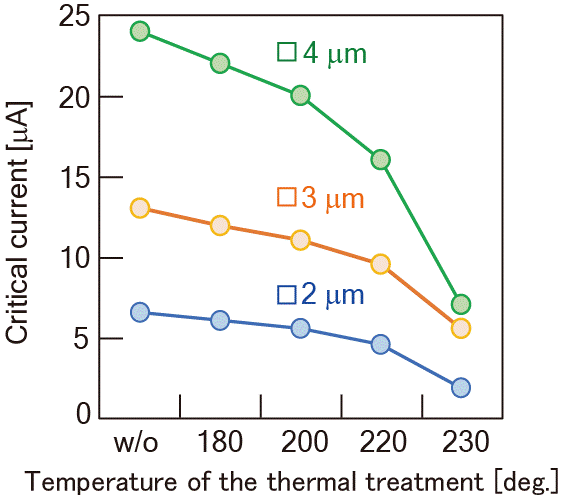


Supplementary Figure S7 | Modulation of the critical current by post-processing with thermal treatment. A Josephson junction (square shape) with a width of 2.5 μm is used in MU1 and MU2.


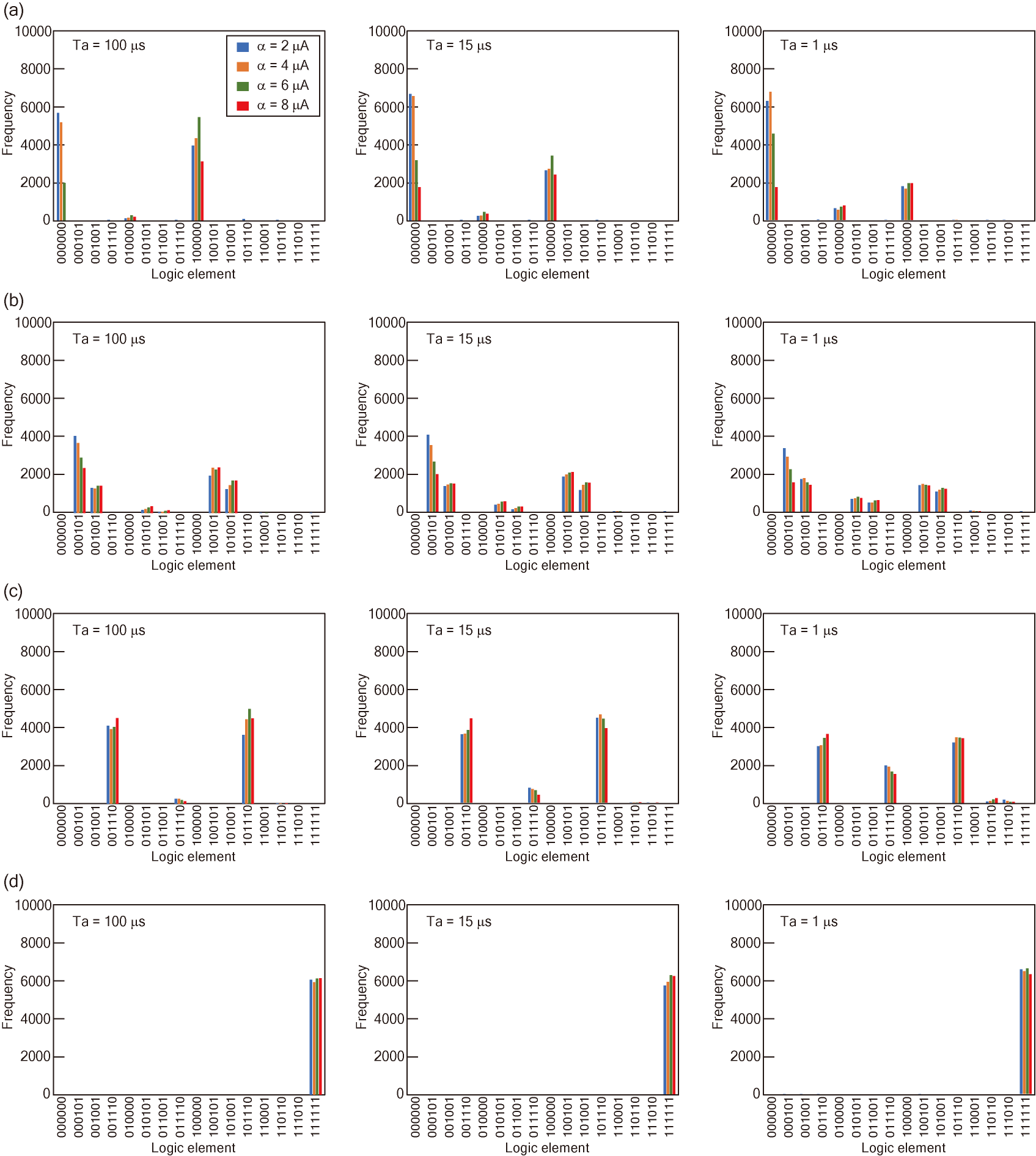


Supplementary Figure S8 | Occupation probabilities of factorized elements for (a) (0,0)_(2)_, (b) (0,1)_(2)_, (c) (1,0)_(2)_, and (d) (1,1)_(2)_ in MU2 under the current conditions at OP1.


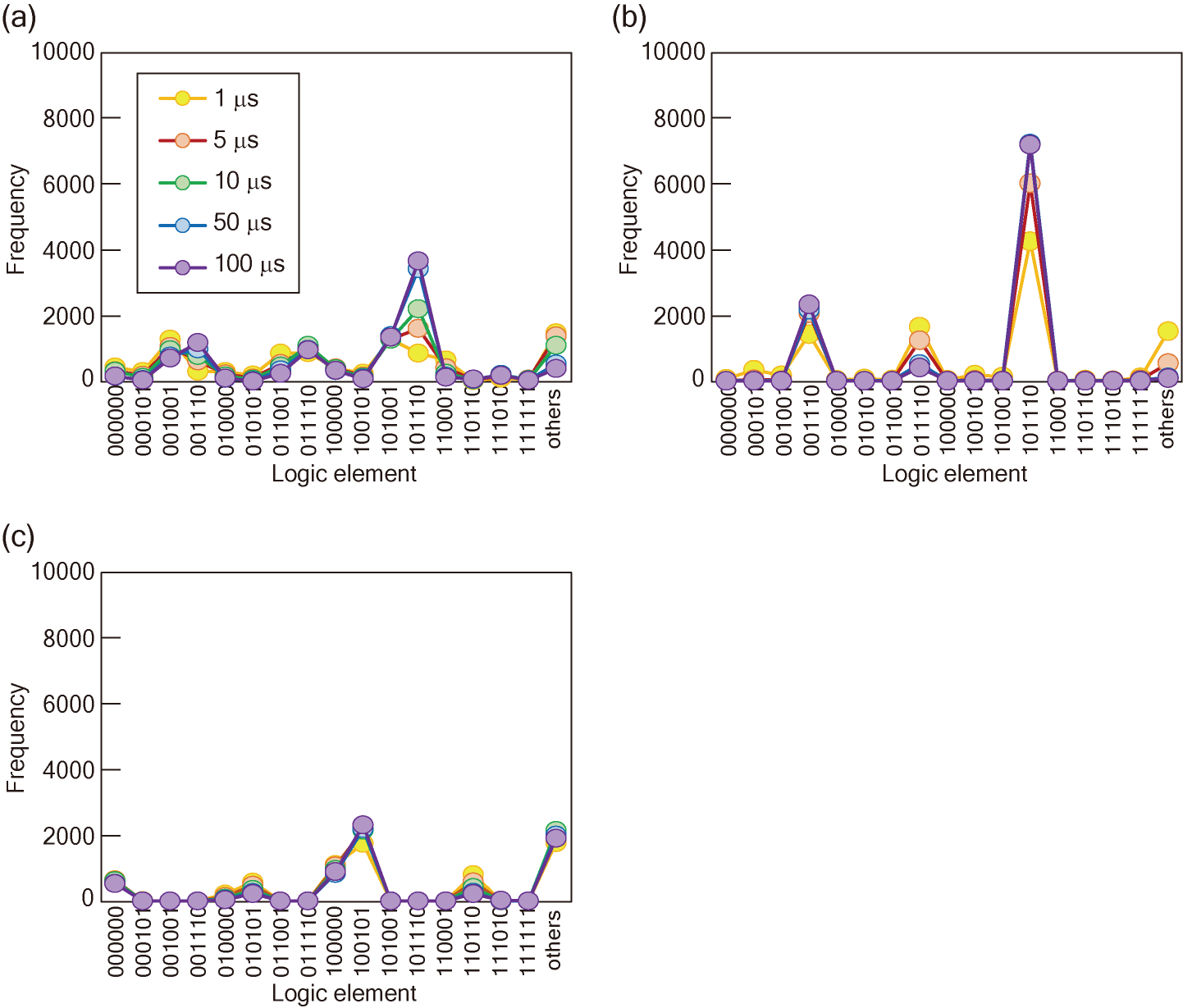


Supplementary Figure S9 | Frequency of each logic element in MU2 obtained at (a) OP2, (b) OP1, and (c) theoretical degeneracy points (OP3).


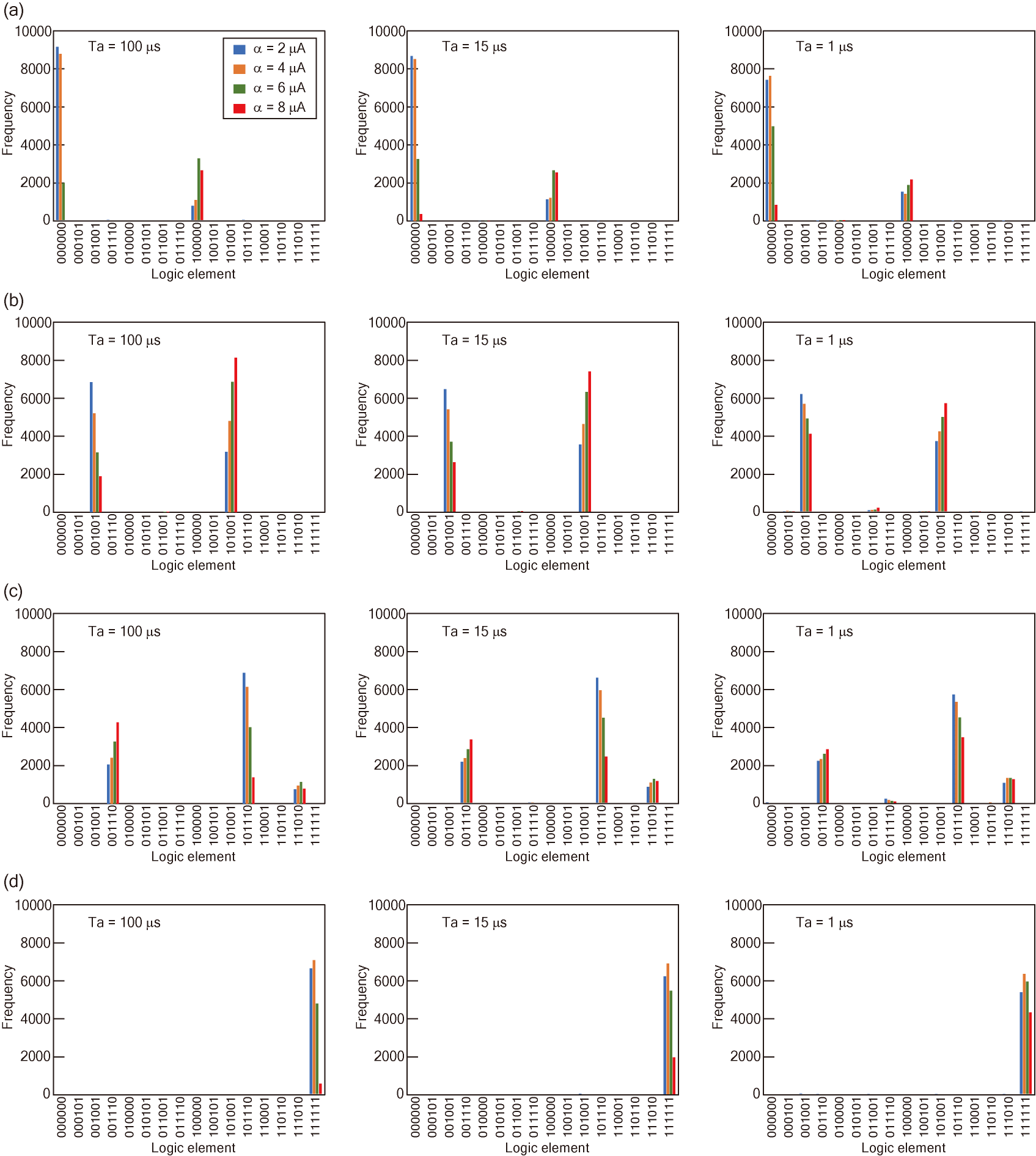


Supplementary Figure S10 | Occupation probabilities of factorized elements for (a) (0,0)_(2)_, (b) (0,1)_(2)_, (c) (1,0)_(2)_, and (d) (1,1)_(2)_ in MU2 under the current conditions at the theoretical degeneracy point.


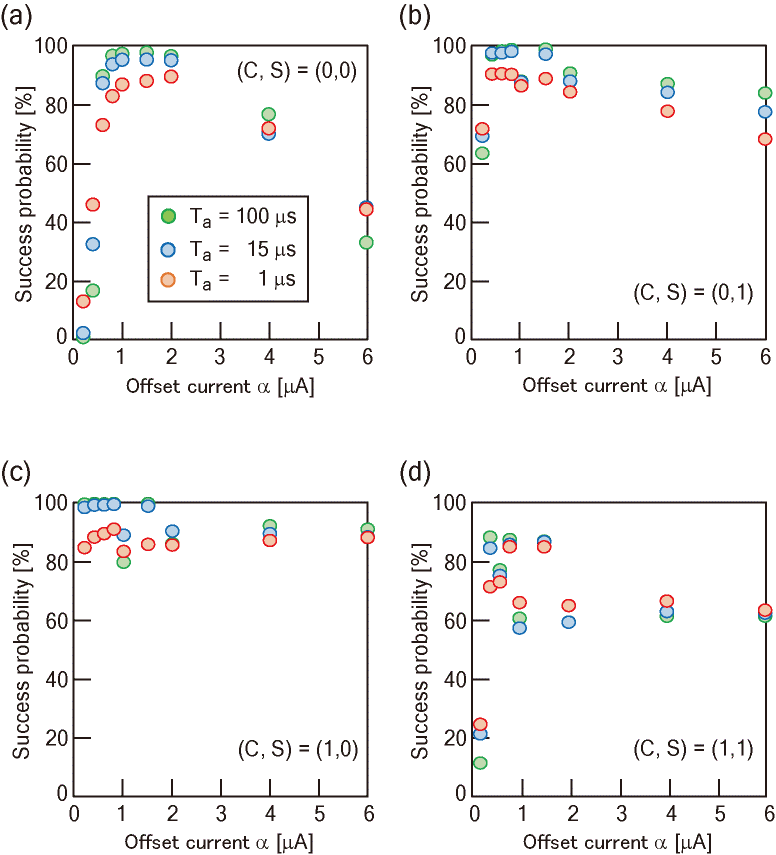


Supplementary Figure S11 | Success probabilities of the factorization carried out with OP1 in MU2 for (a) (0,0)_(2)_, (b) (0,1)_(2)_, (c) (1,0)_(2)_, and (d) (1,1)_(2)_.


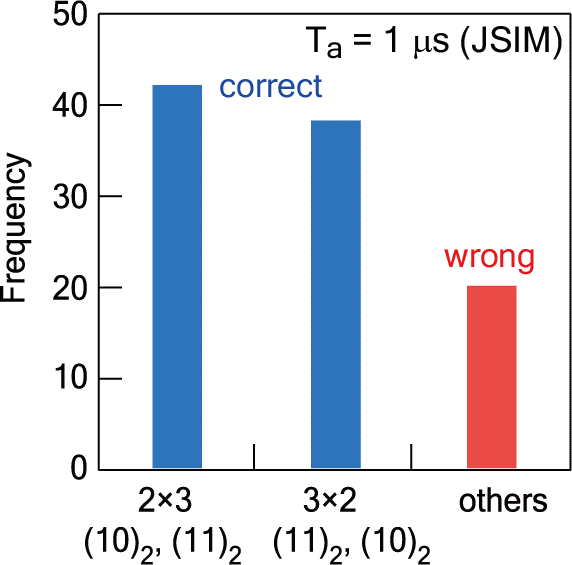


Supplementary Figure S12 | JSIM analysis in a case of factorization of “6”. Simulation is performed with 100 iterations.

**Supplementary References**

1. Johnson M. Amin M. Gildert S. Lanting T. Hamze F. Dickson N. Harris R. Berkley A. Johansson J. Bunyk P. Chapple E. Enderud C. Hilton J. Karimi K. Ladizinsky E. Ladizinsky N. Perminov T. Rich C. Thom M. Tolkacheva E. Truncik C. Uchaikin S. Wang J. Wilson B. and Rose G. Quantum annealing with manufactured spins, *Nature*, **473** 194 (2011).

2. Harris R. Brito F. Berkley A. Johansson J. Johnson M. Lanting T. Bunyk P. Ladizinsky E. Bumble B. Fung A. Kaul A. Kleinsasser A. and Han S. Synchronization of multiple coupled rf-SQUID flux qubits, *New Jour. Phys*., **11** 123022 (2009).

3. Harris R. Lanting T. Berkley A. Johansson J. Johanson M. Bunyk P. Ladizinsky E. Ladizinsky N. Oh T. and Han S. Compound Josephson-junction coupler for flux qubits with minimal crosstalk, *Phys. Rev. B*, **80** 052506 (2009).

4. D-Wave Problem-Solving Handbook (https://docs.dwavesys.com/docs/latest/doc_handbook.html, https://dwavejapan.com/app/uploads/2020/08/09-1171A-C_J-Developer_Guide_Problem_Solving_Handbook.pdf.

5. Maezawa, M., *et al.* Toward Practical-Scale Quantum Annealing Machine for Prime Factoring. *J. Phys. Soc. Jpn*., **88** (2019).

6. Saida, D., Yamanashi, Y., Hidaka, M., Hirayama, F., Imafuku, K., Nagasawa, S. and Kawabata, S. Experimental Demonstrations of Native Implementation of Boolean Logic Hamiltonian in a Superconducting Quantum Annealer. *IEEE Trans. Quant. Eng*., **2** 3103508-3103515 (2021).

7. Boixo S. Smelyanskiy V. Shabani A. Isakov S. Kykman M. Denchev V. Amin M. Smirnov A. Mohseni M. and Neven H. Computational multiqubit tunnelling in programmable quantum annealers, Nature Comm., **7** 10327-10334 (2016).

8. Saida D. Watase N. and Ymanashi Y. Characterization of energy potential in tunable rf-SQUIDs with the classical regime toward precise design of superconducting flux, *Japanese Journal of Applied Physics*, **60** 060906 (2021).

9. Hwang K. Computer Arithmetic : PRINCIPLES, ARCHITECTURE, AND DESIGN.

10. Whitfield J. Faccin M. and Biamonte J. Ground-state spin logic, EPL., **99** 57004 (2012).
